# Supplementary material for: Metal-free atom transfer radical polymerization with ppm catalyst loading under sunlight
Source: Nat Commun. 2021 Jan 18;12:429. doi: 10.1038/s41467-020-20645-8 (PMC7814125; doi:10.1038/s41467-020-20645-8)
Supplement: Supplementary file 1 — Supplementary Information [file 41467_2020_20645_MOESM1_ESM.pdf]

Supplementary Information

Metal-free atom transfer radical polymerization with ppm catalyst loading under sunlight

Ma et al.

Supplementary Methods

General information

Monomers details

Methyl methacrylate (MMA), Benzyl methacrylate (BnMA) and Styrene (St) were purchased from TCI chemicals. 2,2,2-trifluoroethyl methacrylate (TFEMA) was purchased from Adamas chemicals, *n*-butyl acrylate (BA) was purchased from J&K chemicals. MMA was first degassed and dried over CaH_2 overnight, followed by vacuum distillation; then MMA was further purified by titration with neat tri(*n*-octyl)aluminum (Aldrich Chemical) to a yellow end point[1] and distillation under reduced pressure. The monomer was deoxygenized by freeze–pump–thaw cycle three times, stored under a nitrogen atmosphere and sealed up. BnMA was degassed and dried over CaH_2, followed by vacuum distillation. St, TFEMA and BA were purified by passing it through a plug of aluminum oxide (activated, basic) to remove the inhibitor, deoxygenized by freeze–pump–thaw cycle three times, backfilled with argon and sealed up. Subsequently, all of the purified monomers were stored under inert atmosphere at -20℃.

Other chemicals

HPLC-grade dichloromethane (DCM) were first purged with argon and then dried by CaH_2 overnight, followed by vacuum distillation, deoxygenized by freeze–pump–thaw cycle three times, sealed up after adding activated 4 Å molecular sieves. *N*,*N*-dimethylacetamide (DMA, 99.8%, SuperDry, J&K Seal) were purchased from J&K and used as received. Other solvents were purified by vacuum distillation followed by three freeze-pump-thaw cycles and stored under an argon atmosphere before use. The storage life of all the monomers and solvents shall not exceed four weeks.

Ethyl α-bromophenylacetate (EBP) and diethyl 2-bromo-2-methylmalonate (DBMM) were purchased from Alfa Aesar Chemicals and used as received. Ethyl-bromopropanoate (EBrP) and ethyl-2-bromoisobutanoate (EBiB) and Diethyl Bromomalonate (DBM) were purchased from Energy Chemical and used as received. All the initiators were stored in a brown bottle inside a freezer below 5℃.

Unless otherwise specified, all other chemicals and solvents were purchased from Energy chemical, J&K, Adamas or TCI chemicals, and were used as received without further purification.

__Characterization of synthesized organic photocatalysts (OPCs)__

Newly synthesized OPCs were characterized by ^{1}H NMR, ^{13}C NMR, and high-resolution mass spectroscopy. ^{1}H NMR, ^{13}C NMR spectra were recorded using Bruker AVIII 400 spectrometer or AVANCE NEO 600 spectrometer. Chemical shift values were recorded as parts per million (ppm) relative to tetramethylsilane (TMS), chloroform or dichloromethane as internal standard, and coupling constants (*J*) in Hertz. The following abbreviations were used to explain the multiplicities: s = singlet, d = doublet, t = triplet, q = quartet, m = multiplet, br = broad. Mass spectra were recorded on an Agilent Q-TOF 6520 system using electrospray ionization in Positive/Negative ion detection (ESI^+/ESI^-) mode. Significant fragments are reported in the following fashion: m/z (relative intensity).

__Characterization of synthesized polymers__

Newly synthesized polymers were characterized by ^{1}H NMR and gel permeation chromatography (GPC). The number-average molecular weight ($M_{n, GPC}$) and molecular weight distribution (M_w/M_n) values of the obtained polymers determined by a Waters 1515 gel permeation chromatograph (GPC) equipped with a Waters 2414 refractive-index detector, using a Styragel HR 3 THF (7.8 × 300 mm) Column and a Styragel HR 4 THF (7.8 × 300 mm) column with measurable molecular weights ranging from 10^2 to 10^6 g·mol^{-1}. THF was used as eluent at a flow rate of 1.0 mL/min at 35 °C. GPC samples were injected manually and PSS poly(methyl methacrylate) standards were used for calibration. Analysis of some polymer samples' absolute molecular weights was performed via gel permeation chromatography (GPC) coupled with multi-angle light scattering (MALS), using an Agilent HPLC fitted with one guard column and two Shodex GPC KD-806M gel permeation columns, a Wyatt Technology TrEX differential refractometer, and a Wyatt Technology DAWN EOS light scattering detector, using THF as the eluent at a flow rate of 1.0 mL/min. The dn/dc value used for PMMA was 0.084 and the dn/dc value used for PBA was 0.065.

Conversions for homopolymerization and copolymerization are recorded by nuclear magnetic resonance proton spectroscopy or gravimetric methods. The polymer composition was determined using a ^{1}H NMR spectrometer (Bruker AVIII 400 MHz spectrometer or AVANCE NEO 600 MHz spectrometer) with $CDCl_3$ or *d*-DMSO as the solvent.

Matrix assisted laser desorption/ionization time-of-flight mass (MALDI-TOF-MS) spectra were recorded in linear positive ion mode and externally calibrated with "TOF/TOF calibration" standard solution. The acceleration voltage wan set to 19.53 KV and the extraction delay time used was 350 ns. Thin layer method was used by spotting 0.5 µL of PMMA sample (dissolved in THF), 0.5uL of matrix [*trans*-2-3-(4-*tert*-Butylphenyl) 2-methyl-2-propenylidene] malononitrile (DCTB)] on the target plate (ground steel) and allowed to dry at ambient conditions before spectral acquisition.

__Photophysical measurements__

The ultraviolet−visible (UV−vis) spectra were obtained using a Perkins Elmer Lambda 900 spectrometer equipped with a PTP-1 Peltier temperature controller and the photoluminescence (PL) spectra were recorded at room temperature on an Edinburgh Instruments, FLS980 spectrometer equipped with a 450 W Xe lamp for excitation and detected by a photomultiplier (PMT R928P). UV−vis measurements were carried out using anhydrous DCM solution at sample concentration of 0.04, 0.08, 0.12 mM respectively. (Transparent cuvette on four sides: $1\times1\times5$ cm^3); PL measurements were carried out using anhydrous DCM solution at sample concentration of 0.08 mM. Fluorescence decay measurements were carried out by the time-correlated single photon counting (TCSPC) technique. TCSPC event timer with 1 ns time resolution was used to measure the PL decay. The excitation source was a 340nm pulsed light emitting diode (EP-LED Edinburgh Instruments) of pule width (FWHM) 835.5 ps. The decay time fitting procedure was carried out by using the F980 software (Edinburgh Instruments). Smallest residual values were obtained in the fitting procedure.

Electrochemical measurements

Cyclic voltammetry experiments were carried out with a CHI660 D electrochemical workstation (Shanghai Chenhua Instrument Plant, China) using a one compartment electrolysis cell consisting of a typical glassy carbon working electrode (3 mm diameter), a platinum wire counter electrode, and a Ag/AgCl reference electrode. Before performing electrochemical cleaning, the electrode should be sonicated in ethanol and deionized water for 1~3mins respectively to obtain a clean electrode. There is no graininess on the electrode surface when polishing on a Microcloth polishing fleece coated with 1μM and 0.05μM alumina powder (both purchased from Shanghai Chenhua) and the polishing can be stopped. For Step1, scan CV until no obvious oxidation peak existence. Subsequently, CV was swept in a 1 mM $K_3[Fe(CN)_6]$/0.1M KCl solution (sweep rate: 50 mV/s, potential window: -0.1-0.5 V, number of turns: 4). Calculate the potential difference peak potential difference of the last circle of CV, the potential difference is required to be close to 59 mV (optimum: 64 mV, acceptable: 65~72 mV). Specifically, the electrode is a silver wire that is coated with a thin layer of silver chloride and an insulated lead wire connects the silver wire with measuring instrument. The electrode also consists of a porous plug on the one end which will allow contact between the field environment with the silver chloride electrolyte. Saturated potassium chloride is added inside the body of the electrode to stabilize the silver chloride concentration and in this condition the electrode's reference potential is known to be +0.197 V at 25 °C. The measurements were done in 1.0 mM DCM solution with 0.1 M tetrabutylammonium hexafluorophosphate (n-Bu_4NPF_6, TCI chemicals) as supporting electrolyte at a scan rate of 50 mV/s. The redox potential was calibrated after each experiment against the ferrocenium/ferrocenecouple (Fc^+/Fc), which allowed conversion of all potentials to the aqueous saturated calomel electrode (SCE) scale by using $E^0\ (Fc^+/Fc)$ = 0.42 V vs. SCE in CH_3CN.

DFT calculation

We carried out density functional theory, DFT, calculations using the Gaussian09 program package. Geometries optimization calculations were carried out by a meta-GGA hybrid functional PBE0 with 6-31G* basis set for all atoms. Vibrational frequencies were calculated analytically at the same level to obtain the thermodynamic corrections. For details, see: **Computational Details**.

The setup of photocatalytic ATRP polymerization

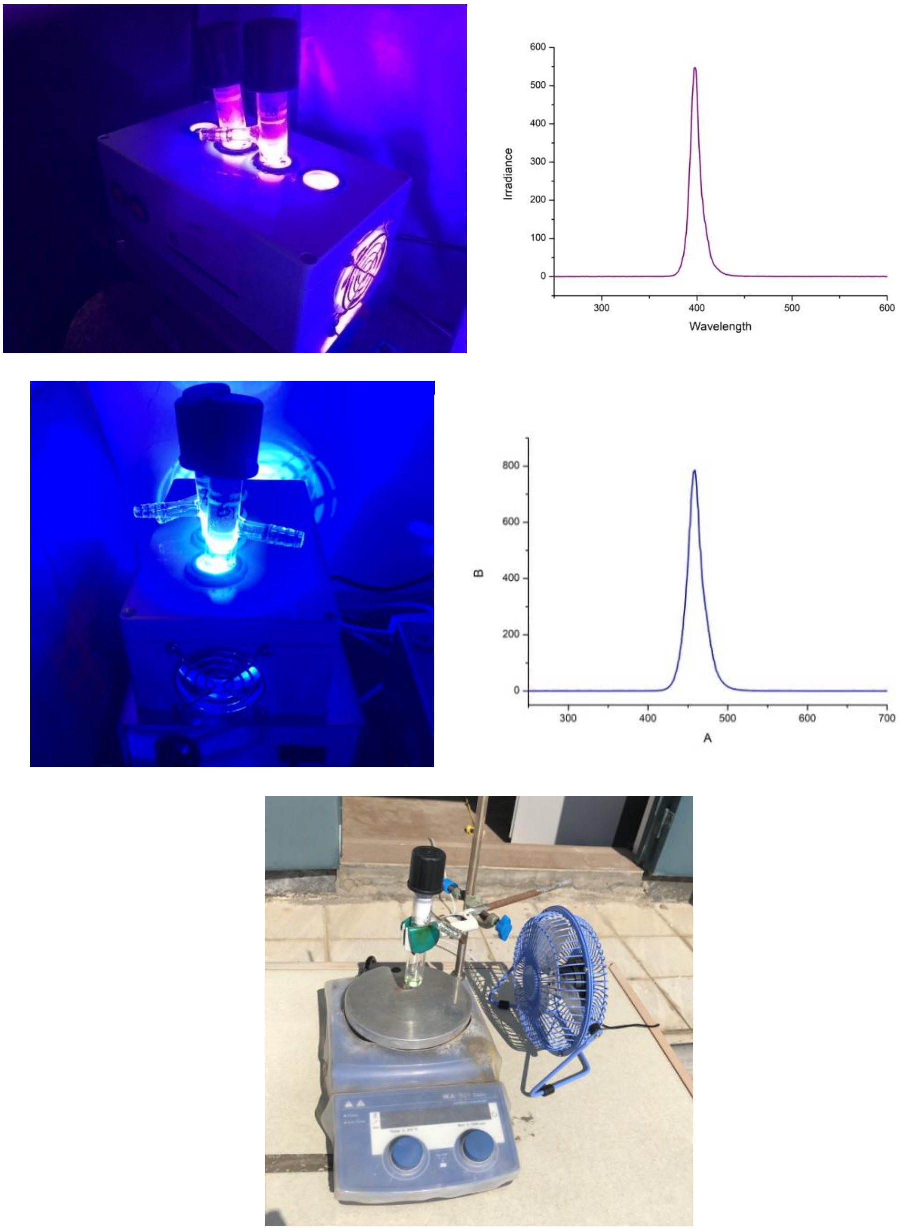

Supplementary Figure 1 | Reactors with 6 W Purple LEDs (λ_{max} = 400 nm), 6 W blue LEDs (λ_{max} = 460 nm) and under sunlight irradiation

6 W purple LEDs and 6 W blue LEDs reactors were purchased from GeAo Chemical (see: www.geaochem.com/) and were used as shown above (Figure S1). All reactions were conducted in a 6 W purple LEDs reactor placed 1 cm from light. At this distance, we estimate the light intensity of 6 W purple LEDs and 6 W blue LEDs to be ~25 mW/cm^2 and ~30 mW/cm^2 respectively.

Synthesis and characterization of photocatalysts

Synthesis of organic photocatalyst 5a-5d[2]

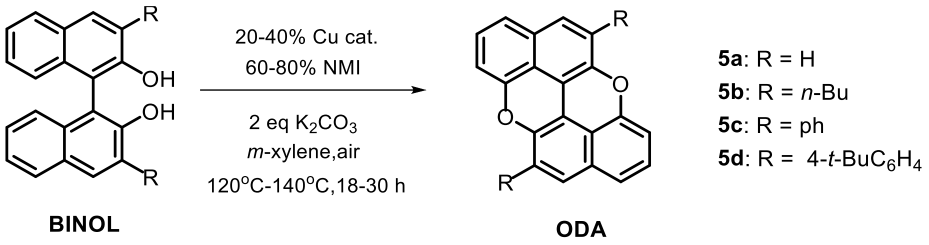

Under ambient air, 1,1'-bi-2-naphthol (143 mg, 0.5 mmol), K_2CO_3 (138 mg, 1 mmol), CuCl(I) (15.0 mg, 0.15 mmol). m-xylene was added (3 mL), followed by N-methylimidazole (24 µL, 0.30 mmol). The vessel was heated at 120 °C for 30 h. After removal of solvent under reduced pressure, the residue was filtered through silica gel short pad with $CHCl_3$ as the eluent and recrystallized from toluene to afford oxygen-dopant of antratherene product ODA **5a** in 75% yield (106 mg) as yellow solid.

xantheno[2,1,9,8-*klmna*]xanthene

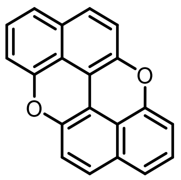

NMR and HRMS data for the catalyst **5a**:

^{1}H NMR (400 MHz, CD_2Cl_2): δ (ppm):7.31 (d, *J* = 8.8 Hz, 2H), 7.09 (m, 4H), 6.90 (d, *J* = 8.8 Hz, 2H), 6.63 (m, 2H).

^{13}C NMR (100 MHz, CD_2Cl_2): δ (ppm): 153.0, 144.6 131.7, 127.6, 126.8, 121.9, 120.5, 117.7, 111.8, 109.0.

HRMS (ESI): *m/z* calculated for $C_{20}H_{10}O_2$: 282.0675, found 282.0674.

5,11-dibutylxantheno[2,1,9,8-*klmna*]xanthene

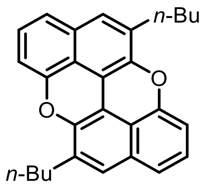

NMR and HRMS data for the catalyst **5b**:

^{1}H NMR (400 MHz, $CDCl_3$): δ (ppm):7.09 – 6.98 (m, 6H), 6.58 (d, *J* = 6.8 Hz, 2H), 2.68 (t, *J* = 6.6 Hz, 4H), 1.68 – 1.64 (m, 4H), 1.45 – 1.40 (m, 4H), 0.98 (t, *J* = 6.4 Hz, 6H).

^{13}C NMR (100 MHz, $CDCl_3$): δ (ppm): 152.8, 143.5, 132.1, 131.4, 127.0, 125.3, 120.5, 119.4, 111.5, 107.7, 32.0, 29.6, 22.6, 14.1.

HRMS (ESI): *m/z* calculated for $C_{28}H_{26}O_2$: 349.1927, found 349.1926.

5,11-diphenylxantheno[2,1,9,8-*klmna*]xanthene

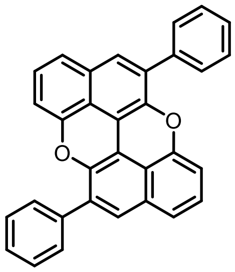

NMR and HRMS data for the catalyst **5c**:

^{1}H NMR (600 MHz, $CDCl_3$): δ (ppm): 7.66 (d, *J* = 7.2 Hz, 4H), 7.49 (t, *J* = 7.5 Hz, 4H), 7.42 (m, 2H), 7.37 (s, 2H), 7.08-7.12 (m, 4H), 6.60 (d, *J* = 7.2 Hz, 2H).

^{13}C NMR (150 MHz, CD_2Cl_2):δ (ppm): 152.5, 142.2, 136.7, 131.4, 131.3, 129.6, 128.4, 127.9, 127.6, 127.2, 121.1, 120.2, 112.4, 108.8.

HRMS (ESI): *m/z* calculated for $C_{32}H_{18}O_2$: 434.1307, found 434.1313.

5,11-bis(4-(*tert*-butyl)phenyl)xantheno[2,1,9,8-*klmna*]xanthene

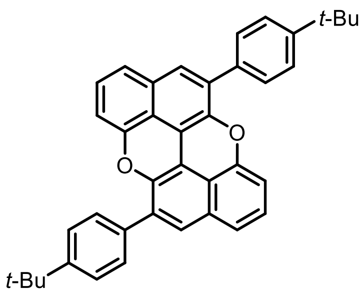

NMR and HRMS data for the catalyst **5d**:

^{1}H NMR (600 MHz, $CDCl_3$): δ (ppm):7.62 (d, *J* = 7.8 Hz, 4H), 7.51 (d, *J* = 8.4 Hz, 4H), 7.37 (s, 2H), 7.07 - 7.11 (m, 4H), 6.62 (dd, *J* = 6.8, 1.5 Hz, 2H), 1.41 (s, 18H).

^{13}C NMR (150 MHz, CD_2Cl_2):δ (ppm): 152.5, 150.9, 142.3, 133.7, 131.4, 131.1, 129.2, 127.5, 127.0, 125.4, 121.0, 120.1, 112.4, 108.7, 34.8, 31.5.

HRMS (ESI): *m/z* calculated for $C_{40}H_{34}O_2$: 546.2559, found 546.2565.

UV-Vis absorption spectra

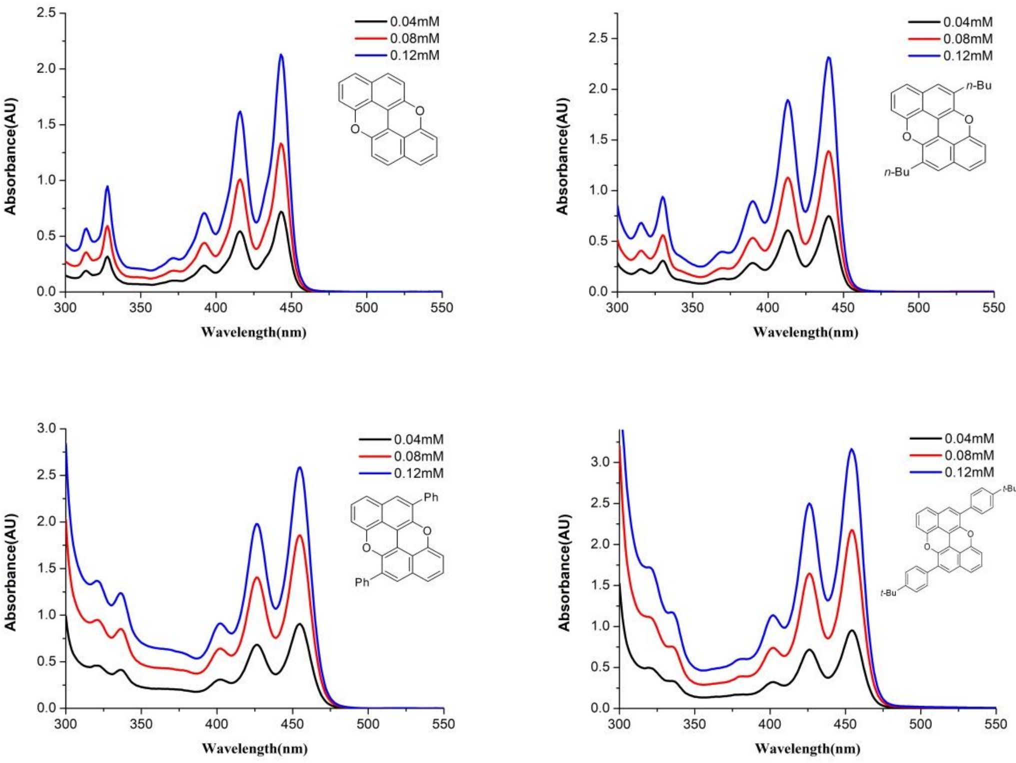

Supplementary Figure 2 | UV-Vis spectra of catalysts 5a, 5b, 5c and 5d at different concentration in DCM.

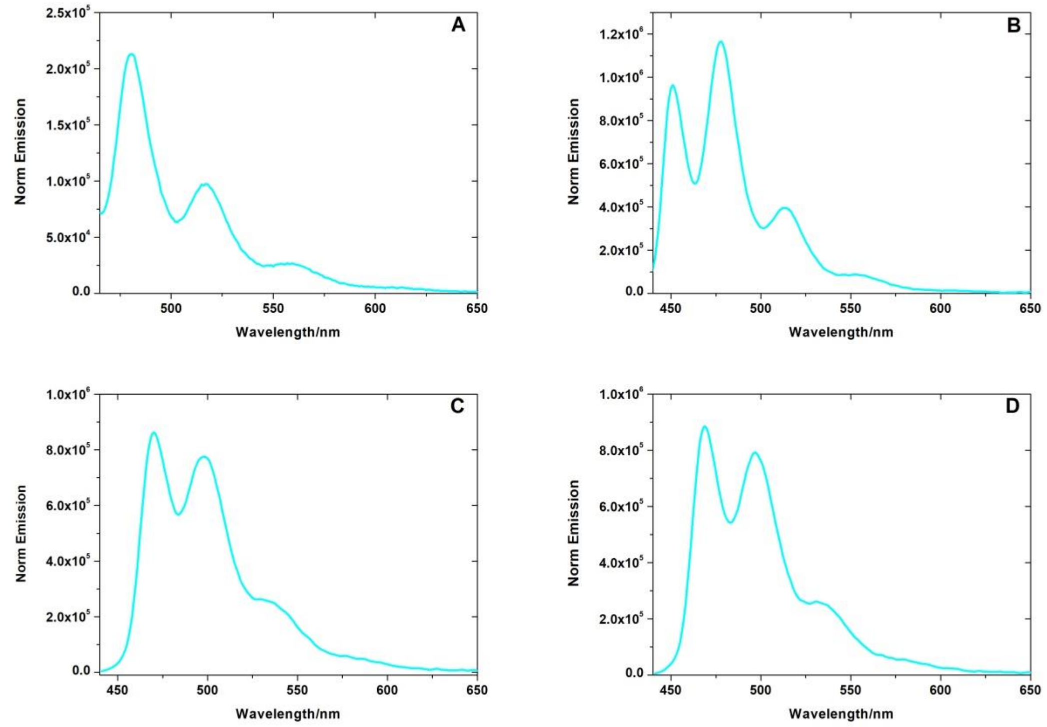

Supplementary Figure 3 | Fluorescence emission spectra of catalyst 5a (A), 5b (B), 5c (C) and 5d (D) in DCM.

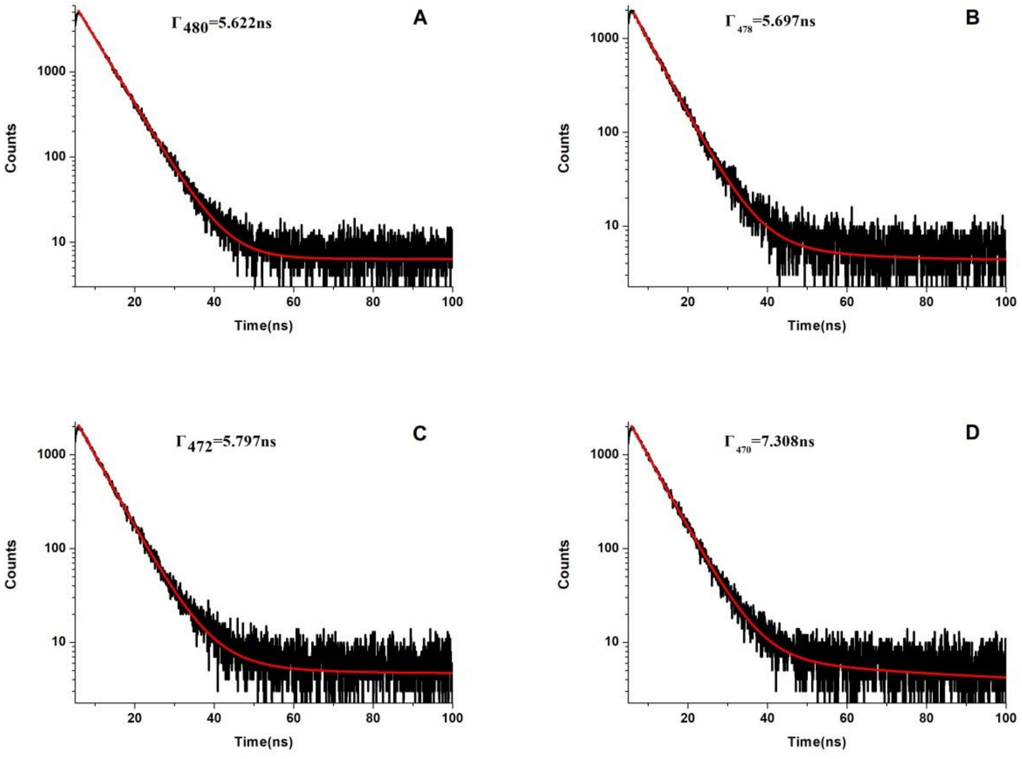

Supplementary Figure 4 | Time-resolved emission decay curves of catalyst 5a (A), 5b (B), 5c (C) and 5d (D) in DCM.

Cyclic voltammetry

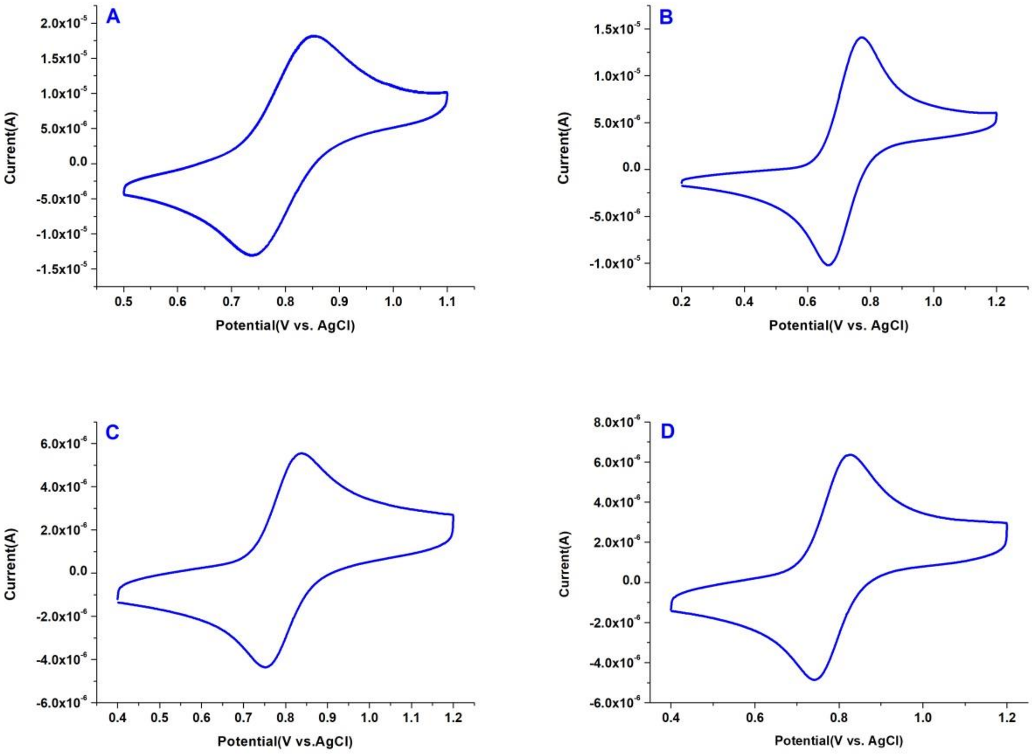

Supplementary Figure 5 | Cyclic voltammograms (vs. Ag/AgCl) of catalyst 5a (A), 5b (B), 5c (C) and 5d (D) in DCM.

Experimental determination of excited state reduction potentials

Using photoluminescence maximum and E^{ox}, the excited state reduction potential was estimated for OPCs (***E*** E_{red} **(OPC$^{•+}$/ OPC*)**) according to the following equations[3]

$$E_{red}\ (OPC^{•+}/\ OPC^*) = E^{ox} - E_{0,0}$$

$$\text{where } E_{0,0} = hc / \lambda_{max} = 1240\ nm / \lambda_{max}$$

Supplementary Table 1. Experimentally measured excited state reduction potentials of 5a-d

Photocatalyst	Abs λ_{max}(nm)	ε λ_{max} (M^{-1} cm^{-1})	em λ_{max}(nm)	E^{ox} vs. SCE	E_{red}($PC^{\cdot+}$/PC*) vs. SCE
**5a**	**443**	**17450**	**481**	**+0.82 V**	**-1.76V**
**5b**	**442**	**18460**	**480**	**+0.74 V**	**-1.84V**
**5c**	**454**	**22580**	**470**	**+0.81 V**	**-1.82V**
**5d**	**455**	**23950**	**469**	**+0.80 V**	**-1.84V**

General method for PMMA synthesis

A typical metal-free organocatalyzed ATRP procedures with the molar ratio of $[MMA]_0$: $[initiator]_0$: $[catalyst]_0$ = 100: 1: 0.05 were showed as follows. The polymerization was conducted with MMA (1.0 mL, 9.35 mmol, 100 eq.) as the model monomer, DBMM (18 μL, 93.5 μmol, 1.0 eq.) as the ATRP initiator, organic photocatalyst (4.70 μmol, 0.5 eq.) and DCM (1.0 mL) as the solvent in a Schlenk tube with a PTFE stirring bar. The mixture was deoxygenized by freeze–pump–thaw cycle three times, backfilled argon and sealed up subsequently. And then the polymerization was occurred under purple LED or blue LED irradiation at room temperature. After the desired time, the tube was opened under argon and 20.0 μL of mixture were syringed out and quenched into $CDCl_3$ containing 250 ppm BHT to determine the monomer conversion by ^1H NMR. The reaction mixture was then diluted with 0.5 mL dichloromethane and dissolved completely, then dripped into 75 mL methanol and stirred for 2 hours. The precipitates was then collected by suction filtration with a Buchner funnel and dried in vacuum oven until a constant weight at 30 °C to give the purified polymers.

For extremely low ppm PC loading experiments, the addition of reagents was conducted in glovebox, other parts as usual. Stock solution of PCs and initiators in dichloromethane were used for a better reproducibility. (Take **5d** as an example: stock solution of **5d**: 1.88 μmol/mL in DCM, weigh 5.10 mg **5d** (9.4 μmol) accurately, dissolve in 5 mL anhydrous dichloromethane, and dilute again if necessary. Second dilution (In chain extension and block polymer experiments of 50/10 ppm PC loading), take 0.5 mL of **5d** solution and dissolve in 4.5 mL of anhydrous dichloromethane to obtain 0.188 μmol/mL pre-prepared solution). All syntheses and manipulations of air- and moisture-sensitive materials were carried out in flamed Schlenk-type glassware on a dual-manifold Schlenk line, on a high-vacuum line, or in an argon-filled glovebox.

General method for other polymers synthesis

A typical ATRP procedure for standard reaction conditions $[monomer]_0$: $[DBMM]_0$: $[\mathbf{5d}]_0$ = [100]: [1]: [0.001] was carried out as follows. A Schlenk tube with a PTFE stirring bar was charged with monomer (6.88 mmol, 100 eq.), DBMM (13.2 μL, 68.8 μmol, 1.0 eq.), PC (0.0688 μmol, 0.001 eq.) and anhydrous DCM as solvent (the 1.2 times volume of solvent as that of the monomer added), inside the glove box. Subsequently, the polymerization was carried out for a certain period of time under blue LED irradiation at room temperature. The tube was opened under argon and 20.0 μL of

mixture were syringed out and quenched into $CDCl_3$ containing 250 ppm BHT to determine the monomer conversion by ^{1}H NMR. To isolate the polymers, reaction mixture was first diluted with 1.0 mL of dichloromethane and dissolved completely, then poured into beaker containing methanol (150 mL) which caused the polymer to precipitate. Subsequent stirring for 2 h, followed by vacuum filtration resulted in dried polymers.

Polymerization procedure for light on-off experiment

Light on-off experiments were performed in glovebox using a [MMA]:[DBMM] ratio of 200:1 with 500 ppm of **5d** and 1 mL:1.5 mL of MMA: DCM. The samples were irradiated for one hour time at which point an aliquot was taken for ^{1}H NMR. The lights were turned off and the reaction vial was wrapped entirely in aluminum foil. After same time of dark period, another aliquot was taken for NMR. The lights were turned back on and the sample was irradiated for two hours, at which point an aliquot was taken for characterization. The lights were turned off and the samples were subjected to another two hour dark period. This light on–off cycle was repeated several times until over 80% conversion of the monomer was achieved.

General methods for analysis of kinetics and molecular weight growth

A typical procedure of kinetics experiments were performed in glovebox using a [MMA]:[DBMM] ratio of 200:1 with 500 ppm **5d** and 1 mL:1.5 mL MMA: DCM. To evaluate the kinetics and growth of molecular weight versus conversion for polymerization, an aliquot of 0.1-0.15 mL of reaction mixture was taken and injected into a solution of $CDCl_3$ containing 250 ppm of the radical inhibitor (BHT), at predetermined times after the start of the polymerization as indicated (when the reaction mixture was exposed to light). Specifically, the operation that each aliquot is taken in this manner to ensure no further introduction of air throughout the polymerization. The aliquot was analysed by ^{1}H NMR spectroscopy to determine the conversion at that time. After NMR analysis, the sample was dried under air, re-dissolved in DCM and dropt into CH_3OH for precipitation, and the M_n and M_w/M_n were analysed by GPC. Analysis of kinetics and molecular weight growth of other catalysts loading can be found in the supplementary details below.

Procedure for PMMA macroinitiator synthesis

MMA (2.00 mL, 18.8 mmol, 100 eq.), DBMM (72 µL, 376 µmol, 2 eq.), and **5d** (5.0 mg, 9.4 µmol, 0.05 eq.) were dissolved in 2.50 mL DCM and reacted according to the above general polymerization procedure for 8 hours. After that, the tube was opened under argon and 20.0 µL of mixture were syringed out and quenched into $CDCl_3$ containing 250 ppm BHT to determine the monomer conversion by ^{1}H NMR (Conv. = 70.4%). At this time, the reaction was removed, poured into 250 mL methanol and stirred for 4 h. The resulting precipitate was then isolated by vacuum filtration and washed with excess methanol. The polymer was then re-dissolved in a minimal amount of DCM again and dripped into 150 mL of methanol and stirred for 2 h to fully remove unreacted monomer, initiator or catalyst. The product was again collected by vacuum filtration and dried under reduced pressure to reveal a slight yellow powder. (M_n = 4.1 kDa, M_w = 4.8 kDa, $Đ$ = 1.18) (GPC trace in **Figure 4.**, black line)

Chain extension and block copolymerization from PMMA macroinitiator

Synthesis of PMMA-*b*-PMMA

A Schlenk tube with a PTFE stirring bar was charged with 0.66 mg of **5d** (1.2×10^{-6} mol, 0.05 eq.) and 99 mg of the PMMA macroinitiator described above (M_n = 4.1 kDa, 1.0 eq.) which were dissolved in 1.0 mL of DCM. Then 0.63 mL of MMA were added (5.87×10^{-3} mol, 243 eq.), reacted according to the above general polymerization procedure for 12 hours. The resulting polymer was isolated according to the above general polymerization procedure and analyzed. After 12 hours, the reaction mixture as loaded into a syringe and slowly dripped into room temperature methanol to precipitate the polymer. After stirring for 4 h, the polymer was collected via vacuum filtration, washed multiple times with excess methanol and dried in vacuum oven until a constant weight at 30 °C to yield 0.578 g of polymer (81% conversion by gravimetric analysis). The resulting chain extended PMMA was found to have M_n = 32.5 kDa, $Đ$ = 1.45. (GPC trace in **Figure 4.**, green line).

Synthesis of PMMA-*b*-PBnMA

A Schlenk tube with a PTFE stirring bar was charged with 0.29 mg of **5d** (0.52×10^{-6} mol, 0.05 eq.) and 43 mg of the PMMA macroinitiator described above (M_n = 4.1 kDa, 1.0 eq.) which were dissolved in 1.50 mL of DCM. Then 0.90 mL of BnMA were added (5.31×10^{-3} mol, 510 eq.), reacted according to the above general polymerization procedure for 14 hours. The resulting polymer was isolated according to the above general polymerization procedure and analyzed. After 14 hours, the reaction mixture as

loaded into a syringe and slowly dripped into room temperature methanol to precipitate the polymer. After stirring for 4 h, the polymer was collected via vacuum filtration, washed multiple times with excess methanol and dried in vacuum oven until a constant weight at 30 °C to yield 0.778 g of polymer (78% yield). The resulting PMMA-b-BnMA copolymer was found to have M_n = 98.2 kDa, $Đ$ = 1.58. (GPC trace in **Figure 4.**, red line).

Synthesis of PMMA-b-PBA

A Schlenk tube with a PTFE stirring bar was charged with 0.25 mg of **5d** (0.427×10^{-6} mol, 0.05 eq.) and 28 mg of the PMMA macroinitiator described above (M_n = 4.1 kDa, 1.0 eq.) which were dissolved in 1.50 mL of DCM. Then 1.05 mL of BA were added (7.29 mmol, 1070 eq.), reacted according to the above general polymerization procedure for 9 hours. The resulting polymer was isolated according to the above general polymerization procedure and analyzed. After 9 hours, the reaction mixture as loaded into a syringe and slowly dripped into room temperature methanol to precipitate the polymer. After stirring for 2 h, a yellow oil crashed out, and the solution was placed into a freezer (ca. -20 °C) for 1 h. The methanol was then decanted off and the residual solvent was removed under reduced pressure. This process was repeated once to yield 0.770 g of a yellow oil (80% yield). The resulting PMMA-*b*-BA) copolymer was found to have M_n = 228 kDa, $Đ$ = 1.63 (GPC trace in **Figure 4.**, blue line).

Supplementary Table 2. Polymerization results of O-ATRP of MMA in various solvents.[a]

Entry	PC	Solvent	Conv	M_n (kDa)	M_w (kDa)	$Đ$
1	**5a**	DMA	88.5%	19.9	26.6	1.34
2	**5a**	DCM	73.3%	12.8	16.0	1.25
3	**5a**	DMF	83.4%	18.3	24.3	1.33
4	**5a**	Toluene	88.1%	17.4	23.0	1.32
5	**5a**	Dioxane	87.3%	19.5	25.9	1.33
6	**5a**	THF	91.2%	21.4	29.3	1.37

[a]Polymerizations of methyl methacrylate were performed at [100]:[1]:[0.05] using EBP as the initiator with purple LED irradiation for 10h and the same volume of solvent as that of the monomer added. (M_n = number-average molecular weight; Mw = weight-average molecular weight). Conv. measured by ^{1}H NMR, M_n and $Đ$ were determined using GPC with PMMA standards.

Supplementary Table 3. Polymerization of MMA with different photocatalysts and initiators.[a]

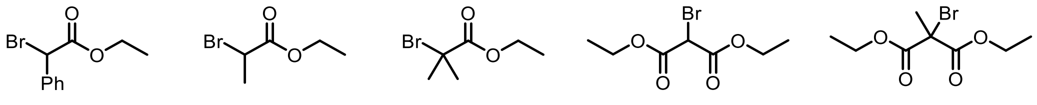

Entry	Initiator	PC	Conv	M_n (kDa)	M_w (kDa)	$Đ$
1	EBP	**5a**	73.3%	12.8	15.8	1.24
2		**5b**	66.5%	12.3	14.6	1.19
3		**5c**	79.2%	13.8	16.8	1.22
4		**5d**	88.2%	13.5	16.6	1.23
5	EBrP	**5a**	72.7%	13.3	16.8	1.27
6		**5b**	59.2%	11.3	12.9	1.15
7		**5d**	76.9%	13.9	17.1	1.23
8[c]	EBiB	**5a**	78.2%	14.9	18.3	1.23
9		**5b**	71.5%	13.9	15.9	1.17
10		**5d**	86.2%	12.8	14.8	1.16
11	DBM	**5a**	80.8%	13.7	16.7	1.22
12		**5b**	60.7%	13.4	15.7	1.17
13		**5d**	78.2%	12.5	11.3	1.16
14	DBMM	**5a**	81.2%	12.8	15.2	1.19
15		**5b**	69.1%	13.5	12.3	1.15
16		**5d**	84.8%	12.0	13.4	1.12

[a] Reaction conditions: MMA (100 equiv), photocatalyst (0.05 equiv), initiator(1.0 equiv), anhydrous DCM (9.4 M of MMA) at room temperature with irradiation from purple LEDs for 10 h. Conv. measured by ^{1}H NMR, M_n and $Đ$ were determined using GPC with PMMA standards.

Supplementary Table 4. Results for polymerization of MMA with various component ratios.[a]

Entry	Ratio	PC	Conv	$M_{n,exp}$	$M_{n,theo.}$	$Đ$
1	500:10:0.5	**5a**	71.2%	6.20	3.82	1.14
2	1000:20:0.5	**5a**	79.5%	7.55	4.23	1.17
3	750:10:0.5	**5a**	75.6%	8.90	5.92	1.19
4	1000:10:0.5	**5a**	81.2%	12.9	8.38	1.19

5	1000:5:0.5	**5a**	66.7%	17.6	13.6	1.30
6	2500:10:1	**5a**	65.2%	22.1	16.5	1.34
7	1000:2:1	**5a**	50.4%	28.6	25.4	1.47
8	500:10:0.5	**5b**	65.9%	6.10	3.55	1.14
9	1000:20:0.5	**5b**	68.3%	5.40	3.67	1.16
10	750:10:0.5	**5b**	54.2%	7.40	4.32	1.17
11	1000:10:0.5	**5b**	69.1%	13.5	7.17	1.15
12	1000:5:0.5	**5b**	67.7%	18.4	13.8	1.38
13	2500:10:1	**5b**	70.5%	22.8	17.8	1.37
14	1000:2:1	**5b**	44.2%	29.5	22.4	1.51
15	500:10:0.5	**5d**	66.4%	5.70	3.57	1.15
16	1000:20:0.5	**5d**	62.5%	7.00	3.38	1.13
17	750:10:0.5	**5d**	76.6%	8.20	6.00	1.13
18	1000:10:0.5	**5d**	84.8%	12.0	8.74	1.12
19	1000:5:0.5	**5d**	73.9%	17.4	15.0	1.25
20	2000:10:0.5	**5d**	64.9%	16.6	13.2	1.27
21	2500:10:1	**5d**	70.8%	22.6	17.9	1.32
22	1000:2:1	**5d**	58.5%	34.9	29.5	1.54
23[b]	1000:0:0.5	**5d**	4.9%	/	40.4	1.53
24[b]	1000:0:0.01	**5d**	3.5%	/	33.2	1.46
25[b]	1000:10:0	**5d**	<2%	/	/	/

[a] Reaction conditions: Photoinduced metal-free ATRP of methyl methacrylate using DBMM as the initiator. Ratio = [MMA]:[DBMM]:[PC]. Solvent: DCM (9.4 M of MMA) at room temperature with irradiation from purple LEDs for 10 h. Conv. measured by ^{1}H NMR, M_n and $Đ$ were determined using GPC with PMMA standards. [b] With 6W blue LEDs, 8 h.

Supplementary Table 5. Polymerization with different ratios at 10 ppm catalyst loading.[a]

Entry	[MMA]:[I]:[PC]	Initiator	PC	Light Source	Time	Conv	$M_{n,GPC}$	$Đ$
1	100:1:0.001	EBP	**5d**	Purple LEDs	11 h	84.6%	15.4	1.28
2	100:1:0.001	EBP	**5d**	Blue LEDs	11 h	90.2%	14.9	1.27
3	1000:5:0.01	DBMM	**5d**	Blue LEDs	12 h	76.7%	20.0	1.26
4	1000:15:0.01	DBMM	**5d**	Blue LEDs	12 h	90.5%	11.0	1.15
5	1000:20:0.01	DBMM	**5d**	Blue LEDs	12 h	93.9%	10.3	1.14

[a] The polymerizations were performed under standard conditions. Conversions were measured by ^{1}H NMR. M_n and $Đ$ were determined using GPC with PMMA standards.

Supplementary Table 6. Polymerization of other monomers.[a]

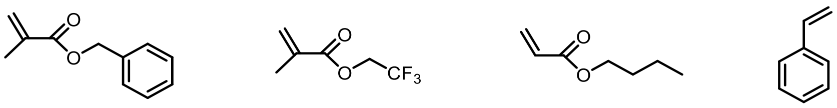

BnMA TFEMA BA St

Entry	Monomer	PC	PC loading	Sol.	Time	Conv	M_n (kDa)	M_w (kDa)	$Đ$
1	BnMA	**5d**	10 ppm	DCM	12h	86.4%	11.0	16.8	1.53
2		**5d**	10 ppm	DMA	12h	97.3%	14.2	22.7	1.61
3	BnMA	**5d**	50 ppm	DCM	12h	93.5%	11.5	14.8	1.29
4		**5d**	50 ppm	DMA	12h	95.1%	14.0	18.0	1.29
5	TFEMA	**5d**	10 ppm	DCM	12h	87.5%	13.6	16.6	1.22
6		**5d**	10 ppm	DMA	12h	88.1%	17.1	19.8	1.16
7	TFEMA	**5d**	50 ppm	DCM	12h	81.6%	11.5	12.5	1.09
8		**5d**	50 ppm	DMA	12h	86.3%	16.2	17.9	1.11
9	BA	**5d**	10 ppm	DCM	7h	78.2%	22.3	33.2	1.49(1.37)
10		**5d**	10 ppm	DMA	7h	88.2%	56.8	92.6	1.63
11	BA	**5d**	50 ppm	DCM	7h	91.6%	30.8	43.4	1.41(1.29)
12		**5d**	50 ppm	DMA	7h	94.8%	40.2	55.1	1.37
13		**5d**	500 ppm	DCM	7h	83.5%	19.2	26.1	1.36(1.19)
14		**5d**	1000 ppm	DCM	7h	99%	28.8	37.1	1.29(1.11)
15	Styrene	**5d**	10 ppm	DCM	36h	38.7%	9.40	33.6	3.58
16		**5d**	10 ppm	DMA	36h	42.6%	8.30	17.7	2.13
17	Styrene	**5d**	50 ppm	DCM	36h	33.9%	13.8	29.7	2.15
18		**5d**	50 ppm	DMA	36h	36.8%	6.47	12.9	1.99

[a] The polymerization of other monomers are initiated by DBMM using **5d** under blue LEDs irradiation. [Monomer]:[DBMM]=100:1. Conv. measured by ^{1}H NMR, M_n and $Đ$ were determined using GPC with PMMA standards. $Đ$ in parenthesis were measured with GPC coupled with MALS.

Supplementary Table 7. Polymerization results of O-ATRP of MMA in various solvents at 10ppm catalyst loading.[a]

Entry	Photocatalyst	Solvent	Conv.	M_n (kDa)	*Đ*	I%
1	**5d**	DMA	81.5%	11.2	1.25	75.2
2	**5d**	DCM	96.9%	12.2	1.19	82.5
3	**5d**	DMF	87.7%	11.8	1.28	76.9
4	**5d**	Toluene	92.5%	10.6	1.34	89.5
5	**5d**	THF	88.2%	11.7	1.34	77.2

[a]Reaction conditions: [MMA]:[DBMM]:[**5d**]= 100:1:0.001(10 ppm) with freshly distilled DBMM under blue LED irradiation. Conv. measured by ^{1}H NMR, M_n and *Đ* were determined using GPC with PMMA standards. I% = ($M_{n,theo}$)/($M_{n,GPC}$) × 100%, $M_{n,theo}$ = [monomer]/[initiator] × M_w of monomer × Conv. % + M_w of initiator.

Supplementary Table 8. A comparison (initiator efficiency) of polymerization of MMA using undistilled (used as received from Alfa Aesar Chemicals) or distilled DBMM.[a]

Entry	PC loading	DBMM	Conv.	$M_{n,theo}$(kDa)	$M_{n,GPC}$ (kDa)	*Đ*	I%[b]
1	(100:1) 500ppm	undistilled	71.8%	7.45	10.9	1.15	68.3
2	(100:1) 500ppm	distilled	86.2%	8.89	10.5	1.18	84.6
3	(100:1) 10ppm	undistilled	90.4%	9.31	14.5	1.17	64.4
4	(100:1) 10ppm	distilled	96.9%	9.96	12.2	1.19	82.5
5	(200:1) 500ppm	undistilled	64.9%	13.2	16.6	1.27	79.6
6	(200:1) 500ppm	distilled	83.8%	17.0	20.3	1.27	83.7
7	(200:1) 10ppm	undistilled	76.7%	15.6	20.0	1.26	78.3
8	(200:1) 10ppm	distilled	93.2%	18.9	20.2	1.29	93.5

[a] Reaction conditions: [MMA]:[DBMM]:[**5d**]= 100:1:x or 200:1:x in DCM at room temperature with irradiation by 460nm blue LEDs in Ar. Solvent: DCM (9.4 M of MMA). Conv. measured by ^{1}H NMR, M_n and *Đ* were determined using GPC with PMMA standards. [b] I %= ($M_{n,theo}$)/($M_{n,GPC}$) × 100%, $M_{n,theo}$ = [monomer]/[initiator] × M_w of monomer × Conv. % + M_w of initiator.

NMR spectra of precipitated polymer products

MMA (2.00 mL, 18.8 mmol, 100 eq.), EBP (65.6 µL, 376 µmol, 2 eq.), and **5d** (9.4 µmol, 0.05 eq.) were dissolved in 2.0 mL DCM and reacted according to the above general polymerization procedure for 8 hours. At this time, the reaction was removed, poured into 150 mL methanol and stirred for 4 h. The resulting precipitate was isolated by vacuum filtration and washed with excess methanol. The polymer was then re-dissolved in a minimal amount of DCM again dripped into 150 mL methanol and stirred for 2 h. The product was again collected by vacuum filtration and dried under reduced pressure to reveal a slight yellow powder. (M_n = 6.90 kDa, $Đ$ = 1.15).

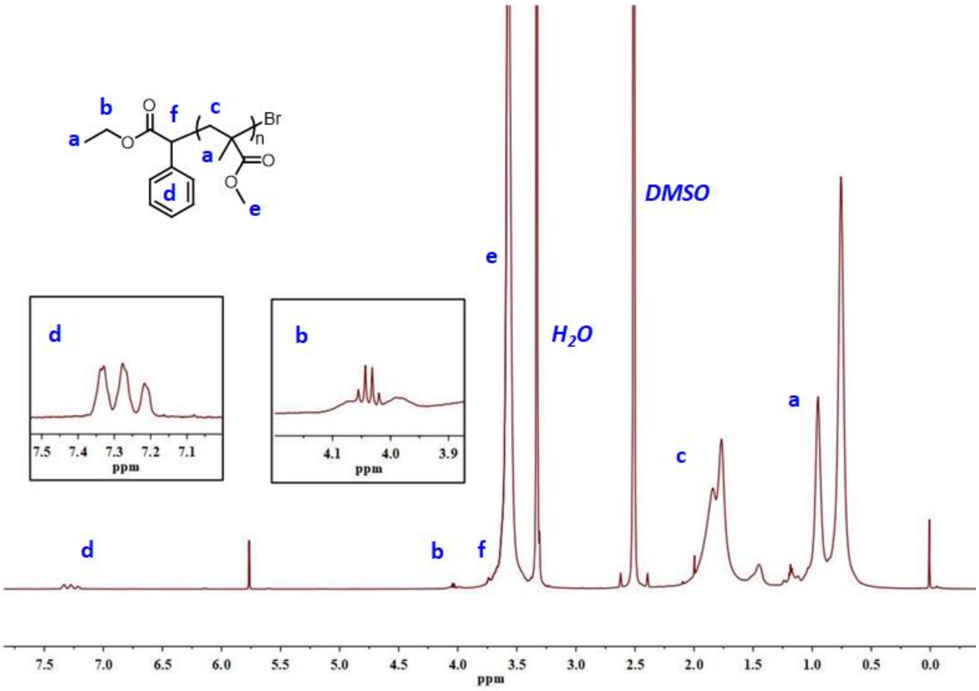

Supplementary Figure 6 | ^{1}H NMR spectrum of isolated poly(methyl methacrylate) (DMSO-*d*6)

Other polymers synthesized Using O-ATRP

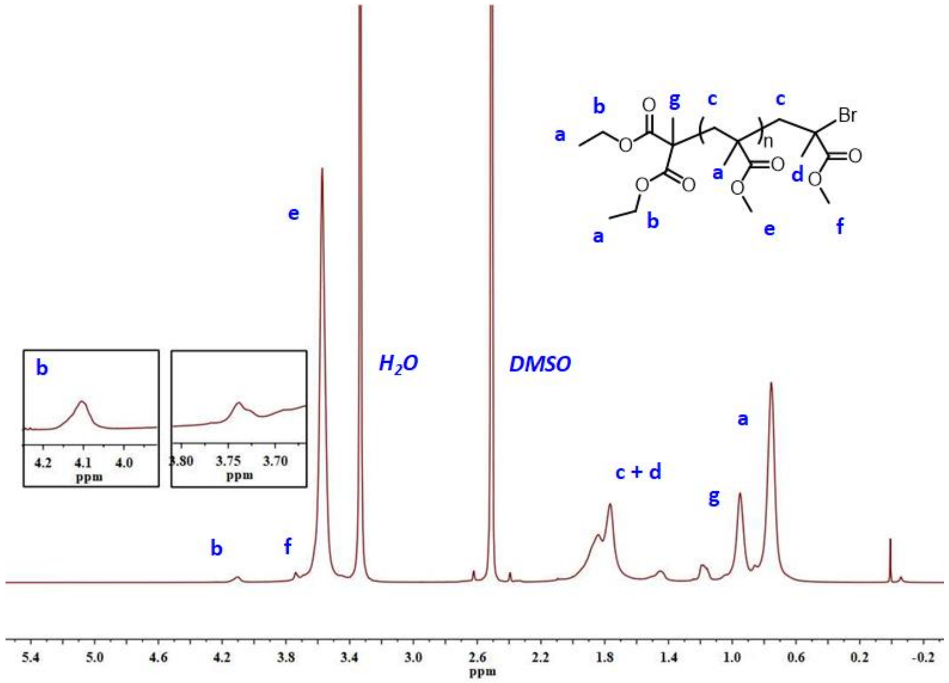

Supplementary Figure 7 | ^{1}H NMR spectrum of isolated poly(methyl methacrylate) made using **5d** (10ppm PC loading) as the catalyst. (DMSO-*d*6)

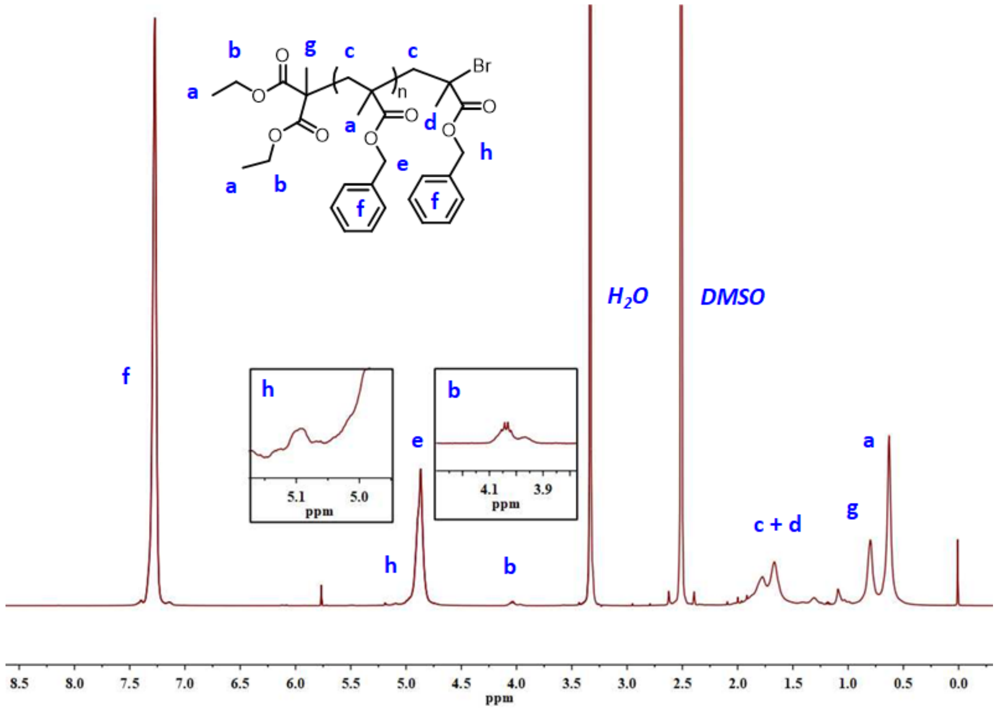

Supplementary Figure 8 | ^{1}H NMR spectrum of isolated poly(benzyl methacrylate) made using **5d** (10 ppm PC loading) as the catalyst. (DMSO-*d*6)

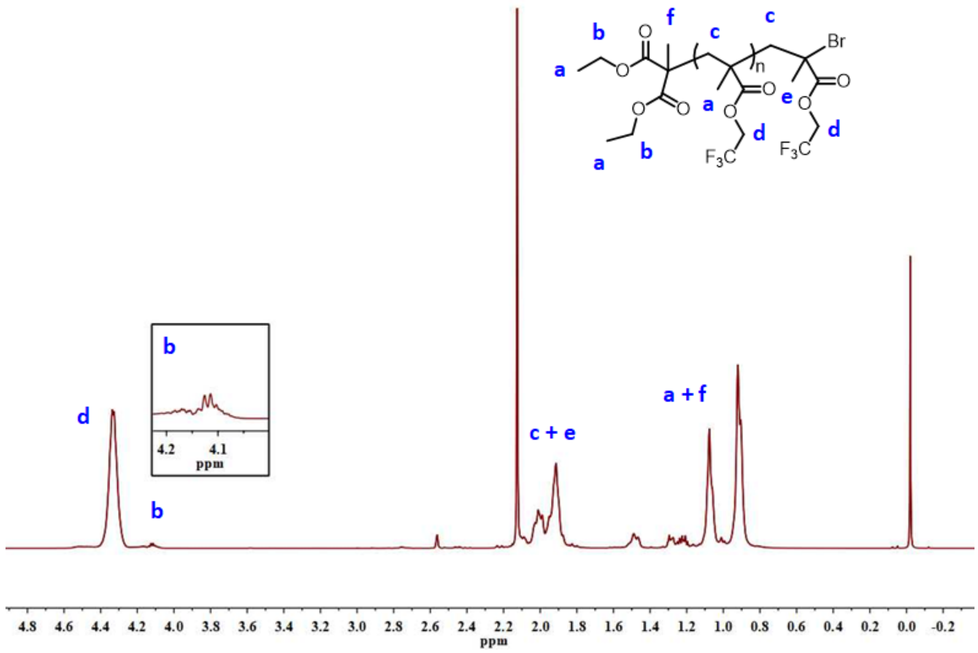

Supplementary Figure 9 | ^{1}H NMR spectrum of isolated poly(2,2,2-trifluoroethyl methacrylate) made using **5d** (10 ppm PC loading) as the catalyst. ($CDCl_3$)

MALDI-TOF analysis of polymer

MMA (1.00 mL, 9.35 mmol, 1000 eq.), DBMM (18.0 µL, 93.5 µmol, 10 eq.), and **5d** (2.5 mg, 9.35 µmol, 0.5 eq.) were dissolved in 1.20 mL DCM and reacted according to the above general polymerization procedure for 10 hours. At this time, the reaction was removed, dripped into 150 mL methanol and stirred for 2 h. The resulting precipitate was then isolated by vacuum filtration and washed with excess methanol. The polymer was then re-dissolved in a minimal amount of DCM again and dripped into 100 mL of methanol and stirred for 1 h to fully remove unreacted monomer, initiator or catalyst. The product was again collected by vacuum filtration and dried under reduced pressure to reveal a slight yellow powder (M_n = 5.20 kDa, $Đ$ = 1.14).

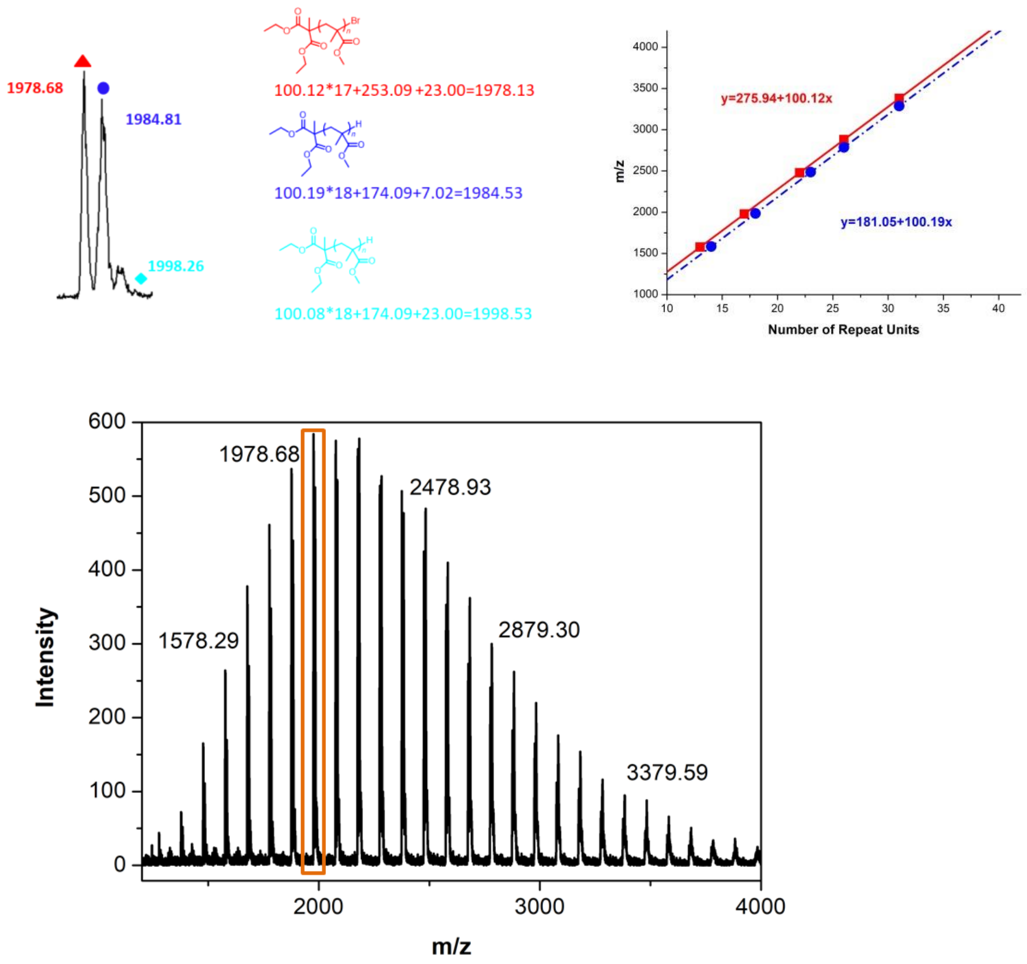

Supplementary Figure 10 | MALDI-TOF mass spectrum of a poly(methyl methacrylate) sample.

Additional chain extension and block copolymerization data

Procedure for PMMA macroinitiator synthesis (50 ppm)

Inside a glovebox, MMA (2.00 mL, 18.8 mmol, 100 eq.), DBMM (72 μL, 376 μmol, 2 eq.), and **5d** (0.5 mg, 0.50 mL, 0.94 μmol, 0.005 eq.) (stock solution of **5d**: 1.88 μmol/mL in anhydrous DCM, 5.1 mg of **5d** (9.4 μmol) in 5.00 mL of anhydrous dichloromethane, diluted again if necessary) were dissolved in 2.00 mL DCM and reacted according to the above general polymerization procedure for 10 hours. The tube was opened under argon and 20.0 μL of mixture were syringed out and quenched into $CDCl_3$ containing 250 ppm BHT to determine the monomer conversion by ^{1}H NMR (Conv. = 88.3%). After that, the reaction was removed, poured into 250 mL methanol and stirred for 4h. The resulting precipitate was then isolated by vacuum filtration and washed with excess methanol. The polymer was then re-dissolved in a minimal amount of dichloromethane again and dripped into 150ml of methanol and stirred for 2h to fully remove unreacted monomer, initiator or catalyst. The product was again collected by vacuum filtration and dried under reduced pressure to reveal a white powder. (M_n = 6.30 kDa, M_w = 7.40 kDa, $Đ$ = 1.19) (GPC trace in **Figure S11**, black line)

Chain extension and block copolymerization from PMMA macroinitiator (50 ppm)

Synthesis of PMMA-*b*-PMMA

Inside a glovebox, a Schlenk tube with a PTFE stirring bar was charged with 0.048 mg of **5d** (8.9×10^{-8} mol, 0.005 eq.) (stock solution of **5d**: 0.188 μmol/mL in anhydrous DCM was used for accurate addition) and 112 mg of the PMMA macroinitiator described above (M_n = 6.30 kDa, 1.0 eq.) which were dissolved in 0.80 mL of DCM. Then 0.40 mL of MMA were added (3.76×10^{-3} mol, 212 eq.), reacted according to the above general polymerization procedure for 10 hours. The resulting polymer was isolated according to the above general polymerization procedure and analyzed. After 10 hours, the reaction mixture as loaded into a syringe and slowly dripped into room temperature methanol to precipitate the polymer. After stirring for 4 h, the polymer was collected via vacuum filtration, washed multiple times with excess methanol and dried in vacuum oven until a constant weight at 30 °C to yield 0.357 g of polymer (65 % conversion by gravimetric analysis). The resulting chain extended PMMA was found to have M_n = 20.7 kDa, $Đ$ = 1.37. (GPC trace in **Figure S11**, red line).

Synthesis of PMMA-*b*-PBnMA

Inside a glovebox, a Schlenk tube with a PTFE stirring bar was charged with 0.055 mg of **5d** (1.0×10^{-7} mol, 0.005 eq.) (stock solution of **5d**: 0.188 μmol/mL in anhydrous DCM was used for accurate addition) and 126 mg of the PMMA macroinitiator described above (M_n= 6.30 kDa, 1.0 eq.) which were dissolved in 1.30 mL of DCM. Then 0.65 mL of BnMA were added (3.8×10^{-3} mol, 191 eq.), reacted according to the above general polymerization procedure for 10 hours. The resulting polymer was isolated according to the above general polymerization procedure and analyzed. After 10 hours, the reaction mixture as loaded into a syringe and slowly dripped into room temperature methanol to precipitate the polymer. After stirring for 4 h, the polymer was collected via vacuum filtration, washed multiple times with excess methanol and dried in vacuum oven until a constant weight at 30 °C to yield 0.477 g of polymer (52% yield). The resulting PMMA-*b*-BnMA copolymer was found to have M_n = 31.8 kDa, $Đ$ = 1.54. (GPC trace in **Figure S11**, green line).

Synthesis of PMMA-*b*-PBA

Inside a glovebox, a Schlenk tube with a PTFE stirring bar was charged with 0.020 mg of **5d** (0.37×10^{-7} mol, 0.005 eq.) (stock solution of **5d**: 0.188 μmol/mL in anhydrous DCM was used for accurate addition) and 47 mg of the PMMA macroinitiator described above (M_n = 6.30 kDa, 1.0 eq.) which were dissolved in 1.2 mL of DCM. Then 0.58 mL of BA were added (4.01×10^{-3} mol, 540 eq.), reacted according to the above general polymerization procedure for 7 hours. The resulting polymer was isolated according to the above general polymerization procedure and analyzed. After 7 hours, the reaction mixture as loaded into a syringe and slowly dripped into room temperature methanol to precipitate the polymer. After stirring for 2 h, a yellow oil crashed out, and the solution was placed into a freezer (ca. -20 °C) for 1 h. The methanol was then decanted off and the residual solvent was removed under reduced pressure. This process was repeated once to yield 0.301 g of a yellow oil (52% yield). The resulting PMMA-*b*-BA) copolymer was found to have M_n = 112 kDa, $Đ$ = 1.54 (GPC trace in **Figure S11**, blue line).

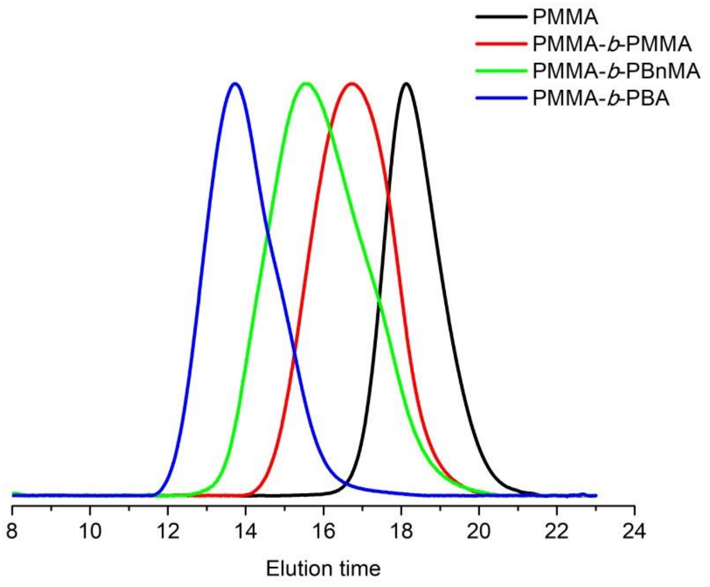

Supplementary Figure 11 | Block polymer preparation. Polymerization of methyl methacrylate performed according to the general polymerization procedure using photoredox catalyst **5d** at 50 ppm catalyst loading under blue LED irradiation. Chain-extension from a PMMA macro-initiator (black) to produce block copolymers with = MMA (red), BnMA (green), and BA (blue).

Procedure for PMMA macroinitiator synthesis (10 ppm)

Inside a glovebox, MMA (2.00 mL, 18.8 mmol, 100 eq.), DBMM (72 μL, 376 μmol, 2 eq.), and **5d** (0.1 mg, 0.1mL, 0.188 μmol, 0.001eq.) (stock solution of **5d**: 1.88 μmol/mL in anhydrous DCM, 5.10 mg of **5d** (9.4 μmol) in 5 mL of anhydrous dichloromethane, diluted again if necessary) were dissolved in 2.50 mL DCM and reacted according to the above general polymerization procedure for 10 hours. The tube was opened under argon and 20.0 μL of mixture were syringed out and quenched into $CDCl_3$ containing 250 ppm BHT to determine the monomer conversion by ^{1}H NMR (Conv. = 82.5%). After that, the reaction was removed, poured into 250 mL methanol and stirred for 4h. The resulting precipitate was then isolated by vacuum filtration and washed with excess methanol. The polymer was then re-dissolved in a minimal amount of dichloromethane again and dripped into 150ml of methanol and stirred for 2h to fully remove unreacted monomer, initiator or catalyst. The product was again collected by vacuum filtration and dried under reduced pressure to reveal a white powder. (M_n = 8.40 kDa, M_w = 10.1 kDa, $Đ$ = 1.20) (GPC trace in **Figure S12**, black line)

Chain extension and block copolymerization from PMMA macroinitiator (10 ppm)

Synthesis of PMMA-*b*-PMMA

Inside a glovebox, A Schlenk tube with a PTFE stirring bar was charged with 0.0107 mg of **5d** (1.97 $\times 10^{-8}$ mol, 0.001 eq.) (OPC, Pre-configured as a dilute solution for easy and accurate addition, stock solution of **5d**: 0.188 µmol/mL in anhydrous DCM) and 165 mg of the PMMA macroinitiator described above (M_n = 8.40 kDa, 1.0 eq.) which were dissolved in 0.90 mL of DCM. Then 0.46 mL of MMA were added (4.33×10^{-3} mol, 220 eq.), reacted according to the above general polymerization procedure for 10 hours. The resulting polymer was isolated according to the above general polymerization procedure and analyzed. After 10 hours, the reaction mixture as loaded into a syringe and slowly dripped into room temperature methanol to precipitate the polymer. After stirring for 4h, the polymer was collected via vacuum filtration, washed multiple times with excess methanol and dried in vacuum oven until a constant weight at 30 °C to yield 0.410 g of polymer (57 % conversion by gravimetric analysis). The resulting chain extended PMMA was found to have M_n = 22.8 kDa, $Đ$ = 1.46. (GPC trace in **Figure S12**, red line).

Synthesis of PMMA-*b*-PBnMA

Inside a glovebox, A Schlenk tube with a PTFE stirring bar was charged with 0.0092 mg of **5d** (1.69 $\times 10^{-8}$ mol, 0.001 eq.) (Stock solution of **5d**: 0.188 µmol/mL in anhydrous DCM was used for accurate addition) and 142 mg of the PMMA macroinitiator described above (M_n= 8.40 kDa, 1.0 eq.) which were dissolved in 1.20 mL of DCM. Then 0.58 mL of BnMA were added (3.4×10^{-3} mol, 202 eq.), reacted according to the above general polymerization procedure for 10 hours. The resulting polymer was isolated according to the above general polymerization procedure and analyzed. After 10 hours, the reaction mixture as loaded into a syringe and slowly dripped into room temperature methanol to precipitate the polymer. After stirring for 4 h, the polymer was collected via vacuum filtration, washed multiple times with excess methanol and dried in vacuum oven until a constant weight at 30 °C to yield 0.425 g of polymer (47% yield). The resulting PMMA-*b*-BnMA copolymer was found to have M_n = 34.2 kDa, $Đ$ = 1.60. (GPC trace in **Figure S12**, green line).

Synthesis of PMMA-*b*-PBA

Inside a glovebox, A Schlenk tube with a PTFE stirring bar was charged with 0.0037 mg of **5d** (0.68×10^{-8} mol, 0.001 eq.) (Stock solution of **5d**: 0.188 μmol/mL in anhydrous DCM was used for accurate addition) and 58 mg of the PMMA macroinitiator described above (M_n = 8.40 kDa, 1.0 eq.) which were dissolved in 1.2 mL of DCM. Then 0.58 mL of BA were added (4.01×10^{-3} mol, 585 eq.), reacted according to the above general polymerization procedure for 7 hours. The resulting polymer was isolated according to the above general polymerization procedure and analyzed. After 7 hours, the reaction mixture as loaded into a syringe and slowly dripped into room temperature methanol to precipitate the polymer. After stirring for 2 h, a yellow oil crashed out, and the solution was placed into a freezer (ca. -20 °C) for 1 h. The methanol was then decanted off and the residual solvent was removed under reduced pressure. This process was repeated once to yield 0.358 g of a yellow oil (59% yield). The resulting PMMA-*b*-BA) copolymer was found to have M_n = 125 kDa, $Đ$ = 1.65 (GPC trace in **Figure S12**, purple line)

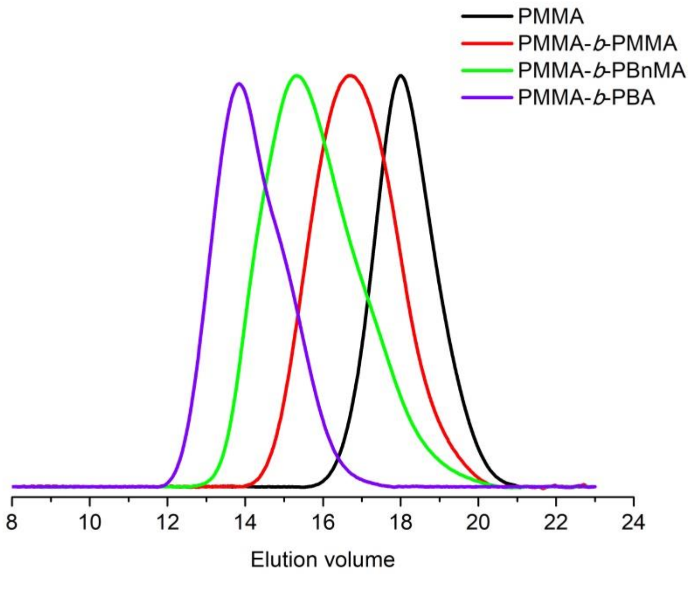

Supplementary Figure 12 | Block polymer preparation. Polymerization of methyl methacrylate performed according to the general polymerization procedure using photoredox catalyst **5d** at 10 ppm catalyst loading under blue LED irradiation. Chain-extension from a PMMA macro-initiator (black) to produce block copolymers with=MMA (red), BnMA (green), and BA (purple).

The preparation of triblock copolymer

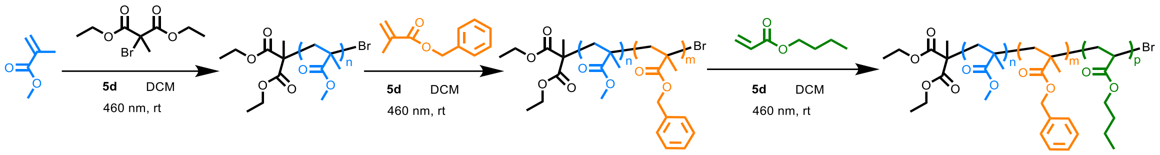

Synthetic route for the synthesis of triblock copolymers (PMMA-*b*-PBnMA-*b*-PBA)

PMMA macroinitiator Synthesis: A PMMA macroinitiator was synthesized using 10 ppm ODA **5d** (stock solution of **5d**: 1.88 μmol/ml in DCM) as PC. Inside a glovebox, MMA (2.00 mL, 18.8 mmol, 100 eq.), DBMM (36 μL, 188 μmol, 1 eq.), and **5d** (0.1 mg, 100 μL, 0.188 μmol, 0.001eq.) were dissolved in 2.50 mL DCM and reacted according to the above general polymerization procedure for 15 hours under the irradiation of blue LED. The tube was opened under argon and 20.0 μL of mixture were syringed out and quenched into $CDCl_3$ containing 250 ppm BHT to determine the monomer conversion by ^{1}H NMR (Conv. = 96%). After that, the reaction was removed, poured into 250 mL methanol and stirred for 5 h. The resulting precipitate was then isolated by vacuum filtration and washed with excess methanol. The polymer was then re-dissolved in a minimal amount of dichloromethane again and dripped into 150 mL of methanol and stirred for 2 h to fully remove unreacted monomer, initiator or catalyst. The polymer was dried to constant weight under vacuum at 30 °C to give a white powder. (M_n = 14.8 kDa, M_w = 17.8 kDa, *Đ* = 1.20) (GPC trace in **Figure S13**, blue line)

Synthesis of PMMA-*b*-PBnMA: Inside a glovebox, A Schlenk tube with a PTFE stirring bar was charged with **5d** (0.38 ×10^{-7} mol, 50 ppm, 0.005 eq.) (Stock solution of **5d**: 0.188 μmol/mL in anhydrous DCM was used for accurate addition) and 115 mg of the PMMA macroinitiator described above (M_n = 14.8 kDa, 1.0 eq.) which were dissolved in 1.00 mL of DCM. Then 276 μL of BnMA were added (1.63×10^{-3} mol, 210 eq.), reacted according to the above general polymerization procedure for 7 hours. The resulting polymer was isolated according to the above general polymerization procedure and analyzed. After 7 hours, the reaction mixture as loaded into a syringe and slowly dripped into methanol to precipitate the polymer. After stirring for 4 h, the polymer was collected via vacuum filtration, washed multiple times with excess methanol and dried in vacuum oven until a constant weight at 30 °C to yield 0.270 g of polymer (54% yield). The resulting PMMA-*b*-BnMA copolymer was found to have M_n = 39.5 kDa, *Đ* = 1.51. (GPC trace in **Figure S13**, orange line).

Synthesis of PMMA-*b*-PBnMA-*b*-PBA: Inside a glovebox, A Schlenk tube with a PTFE stirring bar was charged with **5d** (0.28×10^{-7} mol, 50 ppm, 0.005 eq.) (Stock solution of **5d**: 0.188 μmol/mL in anhydrous DCM was used for accurate addition) and 225 mg of the PMMA-*b*-PBnMA macroinitiator described above (M_n= 39.5 kDa, 1.0 eq.) which were dissolved in 0.80 mL of DCM. Then 147 μL of BA were added (1.02×10^{-3} mol, 180 eq.), reacted according to the above general polymerization procedure for 4 hours. The resulting polymer was isolated according to the above general polymerization procedure and analyzed. After 4 hours, the reaction mixture as loaded into a syringe and slowly dripped into methanol to precipitate the polymer. After stirring for 2 h, a thick polymer crashed out, and the solution was placed into a freezer (ca. -20 °C) for 1 h. The methanol was then decanted off and the residual solvent was removed under reduced pressure. to yield 0.279 g of polymer (42% yield). The resulting PMMA-*b*-BnMA-*b*-PBA copolymer was found to have M_n = 60.6 kDa, $Đ$ = 1.46. (GPC trace in **Figure S13**, green line).

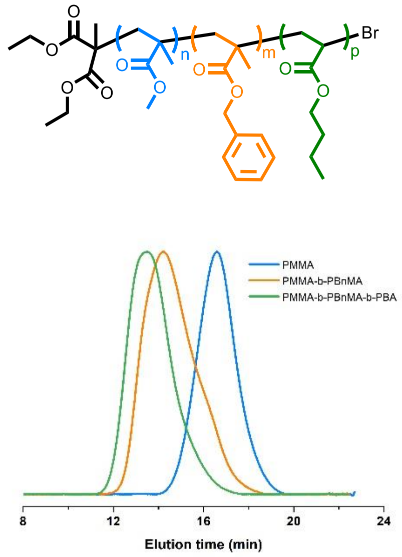

Supplementary Figure 13 | triblock copolymer PMMA-*b*-PBnMA-*b*-PBA. Measured using GPC with PMMA standards.

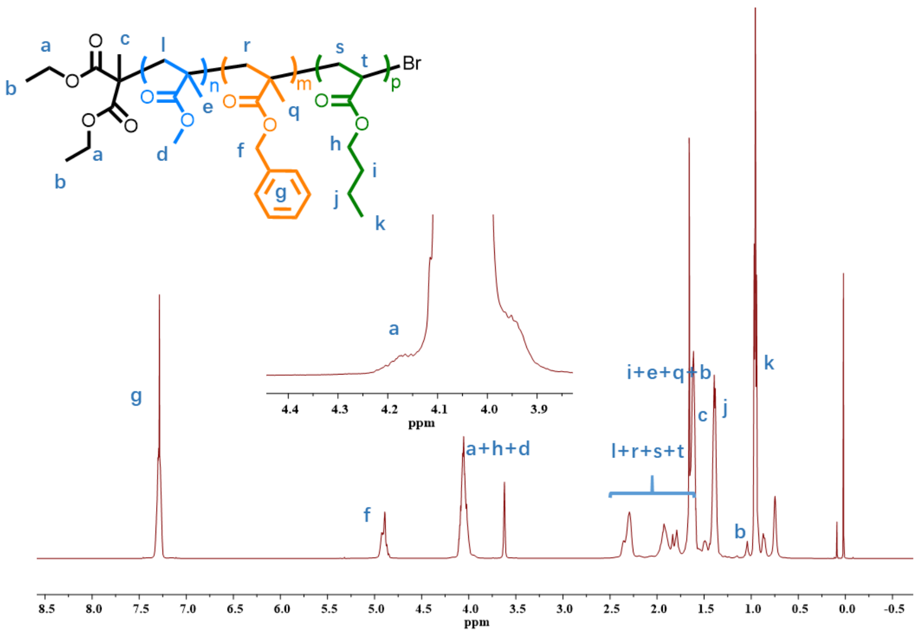

Supplementary Figure 14 | ^{1}H NMR spectrum of PMMA-*b*-PBnMA-*b*-PBA in CDCl$_3$.

NMR spectra of photocatalysts

5a:

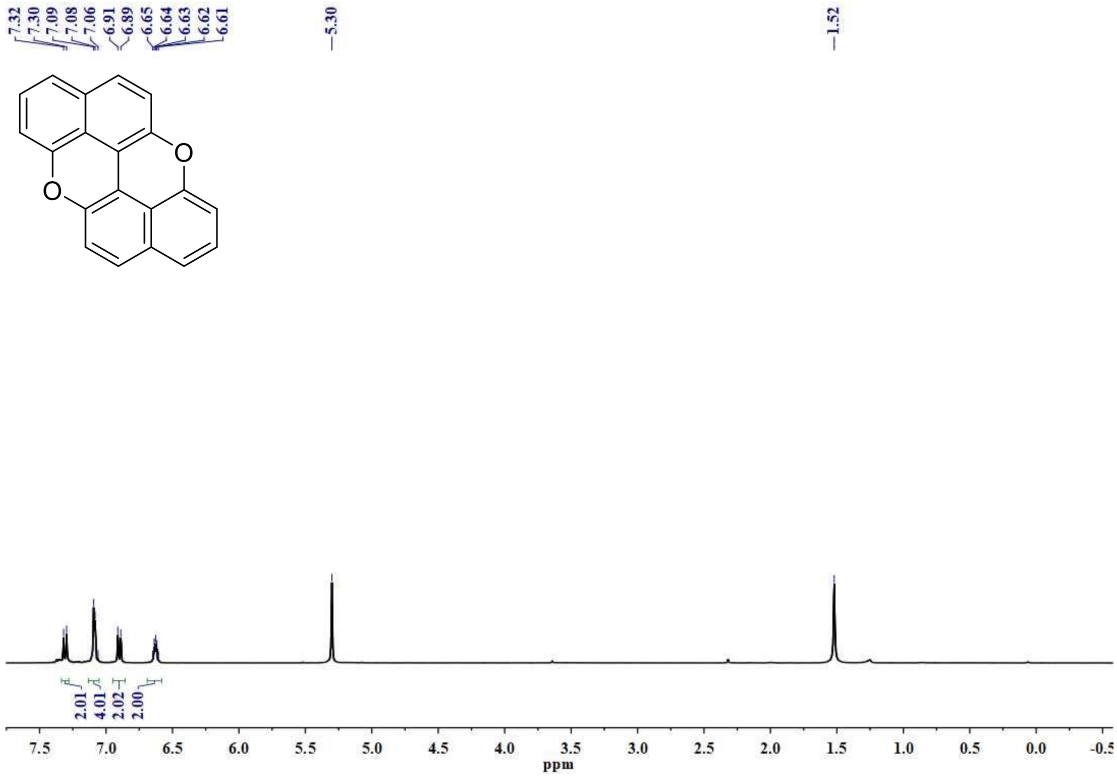

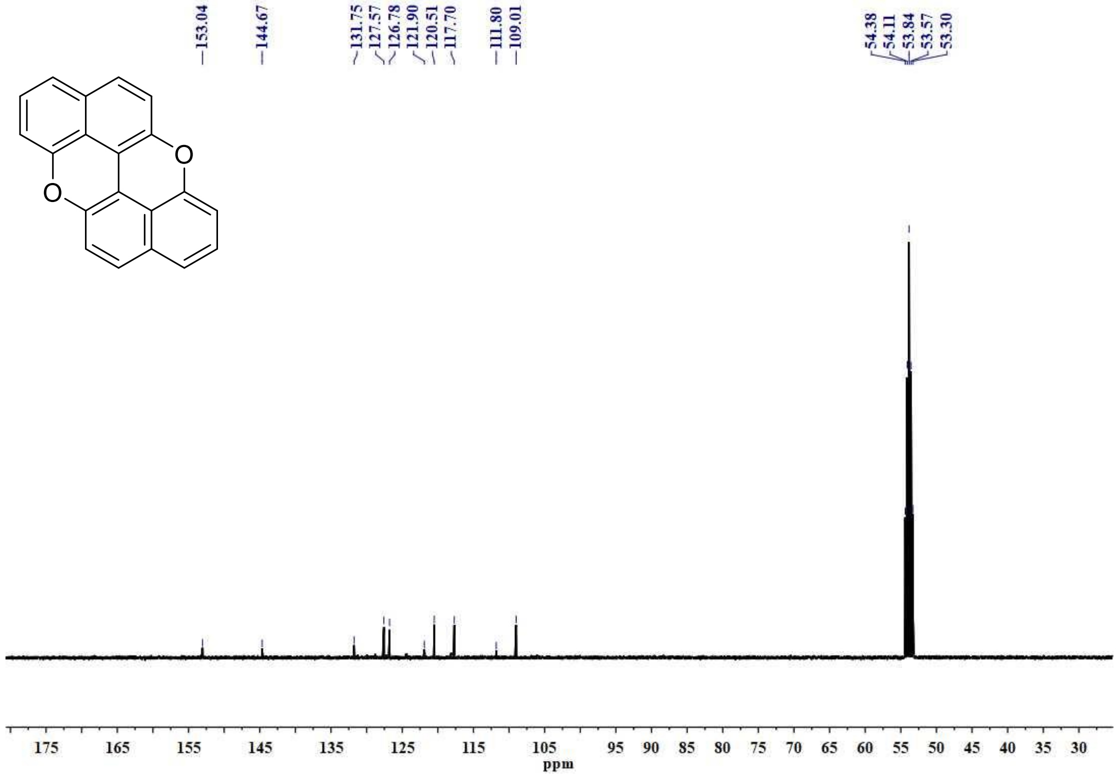

5b:

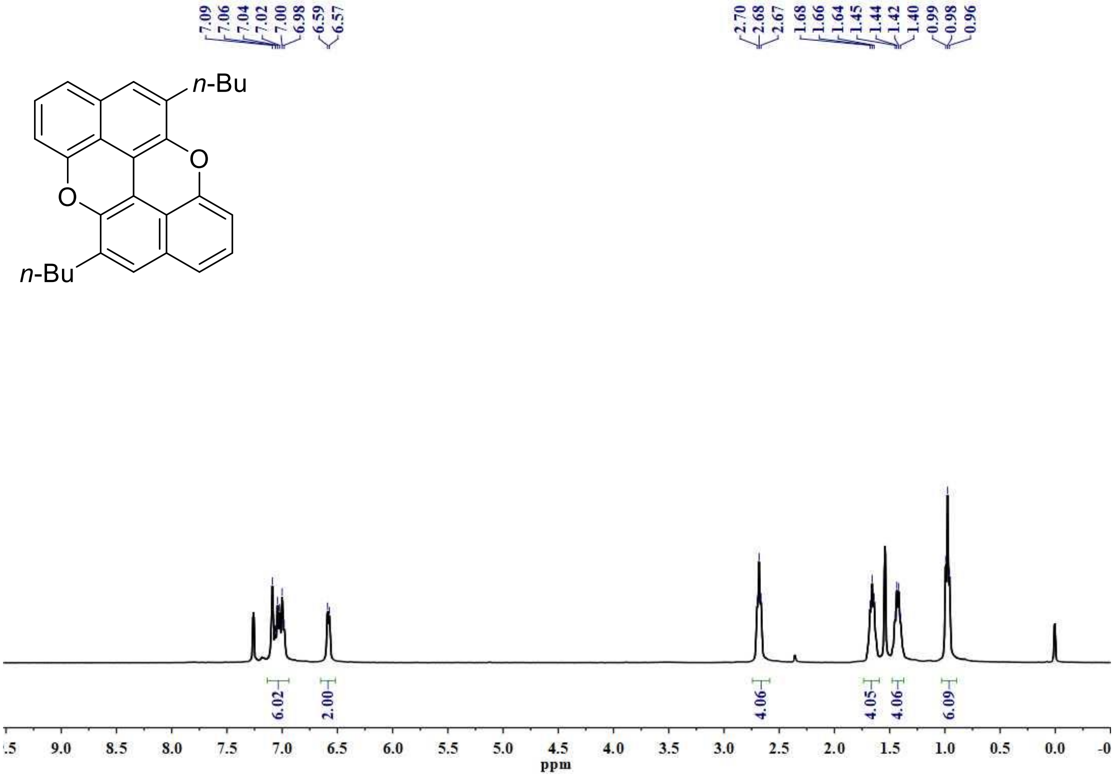
7.09
7.06
7.04
7.02
7.00
6.98
6.59
6.57
2.70
2.68
2.67
1.68
1.66
1.64
1.45
1.44
1.42
1.40
0.99
0.98
0.96
n-Bu
O
O
n-Bu
6.02
2.00
4.06
4.05
4.06
6.09
ppm

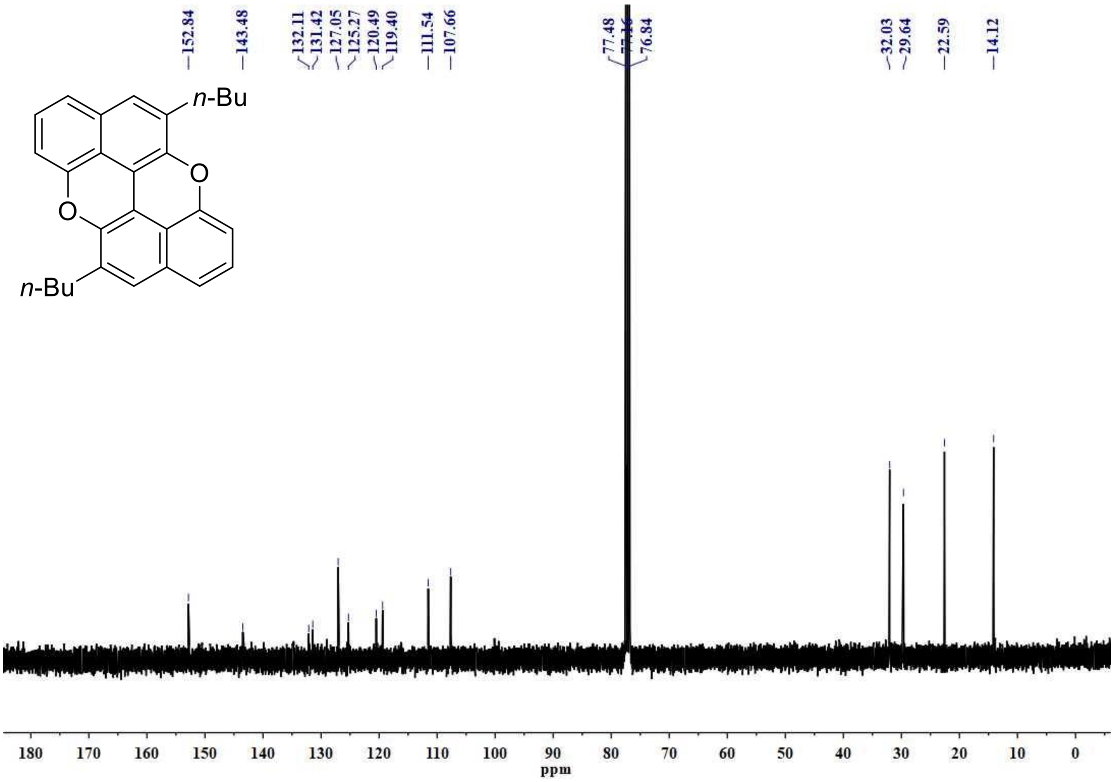
152.84
143.48
132.11
131.42
127.05
125.27
120.49
119.40
111.54
107.66
77.48
76.84
32.03
29.64
22.59
14.12
n-Bu
O
O
n-Bu
ppm

5c:

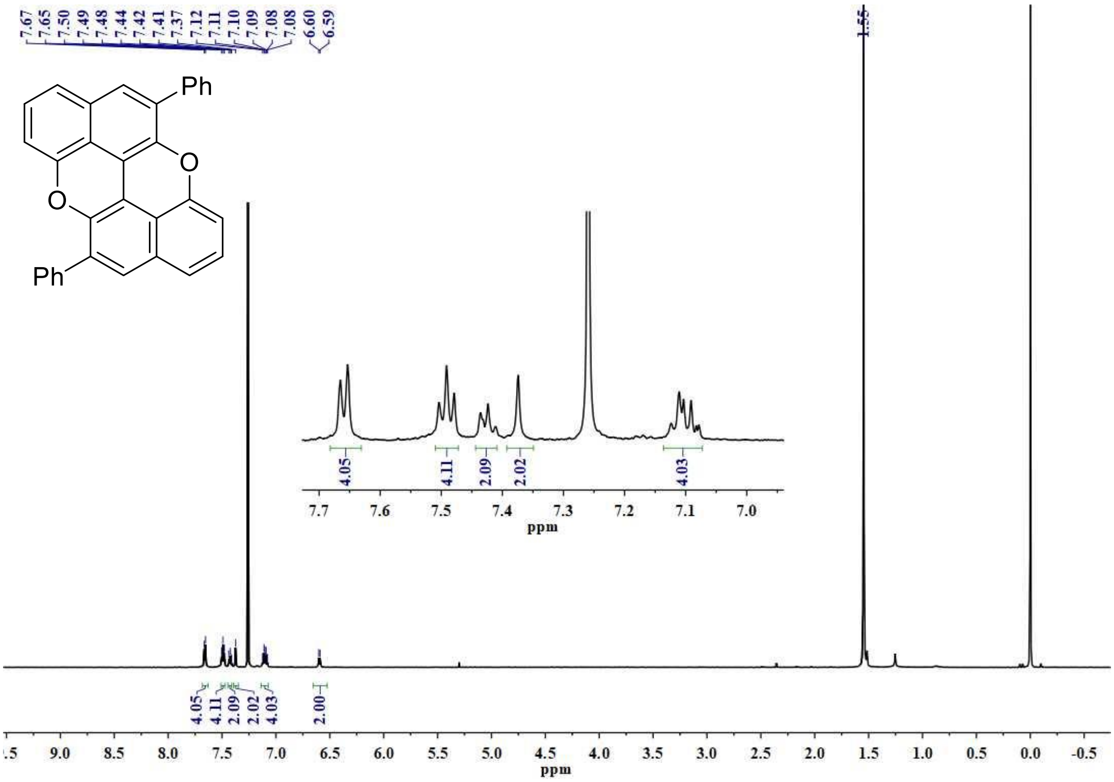
7.67
7.65
7.50
7.49
7.48
7.44
7.42
7.41
7.37
7.12
7.11
7.10
7.09
7.08
7.08
6.60
6.59
1.55
Ph
O
O
Ph
4.05
4.11
2.09
2.02
4.03
2.00
ppm

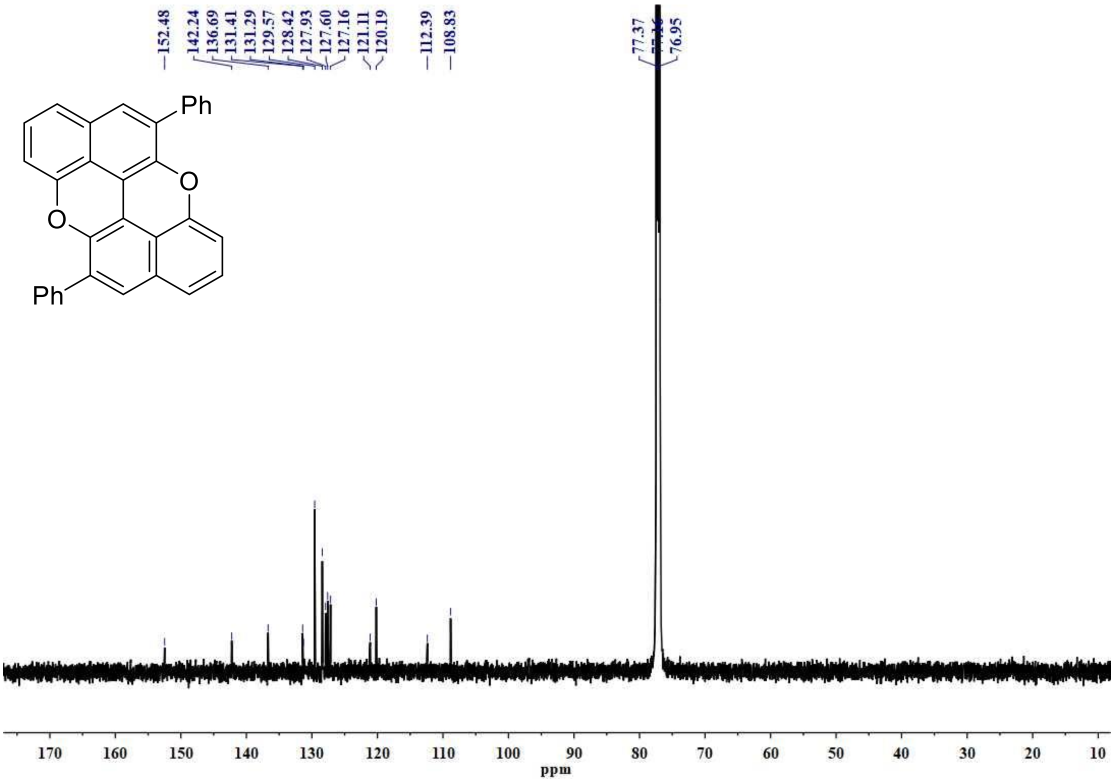
152.48
142.24
136.69
131.41
131.29
129.57
128.42
127.93
127.60
127.16
121.11
120.19
112.39
108.83
77.37
76.95
Ph
O
O
Ph
ppm

5d:

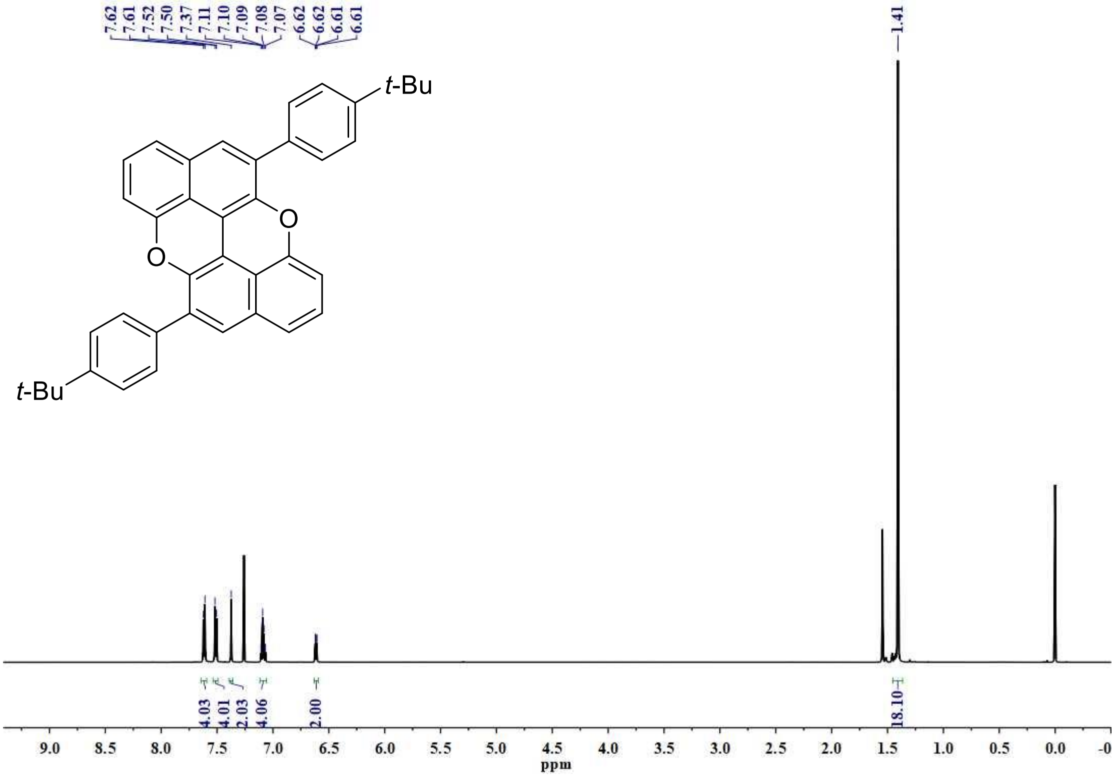
7.62
7.61
7.52
7.50
7.37
7.11
7.10
7.09
7.08
7.07
6.62
6.62
6.61
6.61
1.41
t-Bu
O
O
t-Bu
4.03
4.01
2.03
4.06
2.00
18.10
9.0 8.5 8.0 7.5 7.0 6.5 6.0 5.5 5.0 4.5 4.0 3.5 3.0 2.5 2.0 1.5 1.0 0.5 0.0 -0
ppm

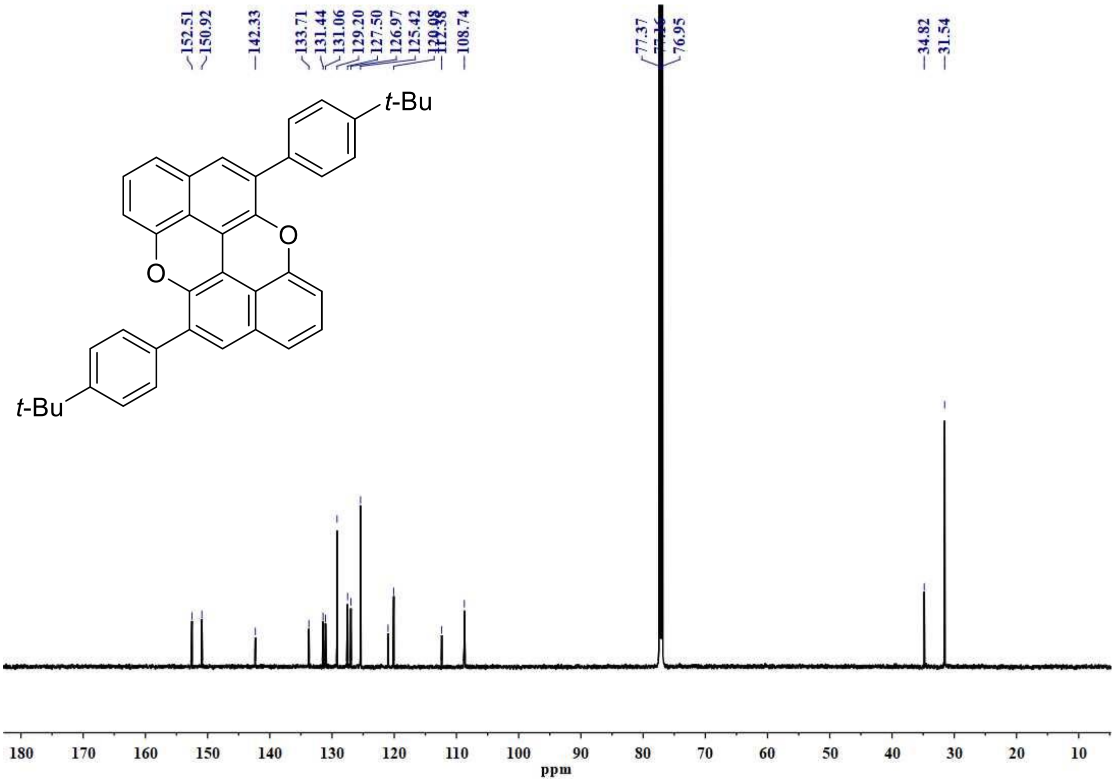
152.51
150.92
142.33
133.71
131.44
131.06
129.20
127.50
126.97
125.42
108.74
77.37
76.95
34.82
31.54
t-Bu
O
O
t-Bu
180 170 160 150 140 130 120 110 100 90 80 70 60 50 40 30 20 10
ppm

Fluorescence quenching study

The solutions of ODA **5a** were excited at 449 nm and the fluorescence spectra were recorded between 465 and 650 nm. The emission of a 0.20 mM solution of **5a** in DCM was measured at varying volumes of diethyl 2-bromo-2-methylmalonate (DBMM, 500 mM). As shown in Figures S15 a significant fluorescence quenching by addition of DBMM was observed. The emission of a 0.20 mM solution of **5a** in DCM was also measured at varying volume of methyl methacrylate (MMA) (1 M). As shown in Figures S18 no significant quenching of the emission of **5a** was observed.

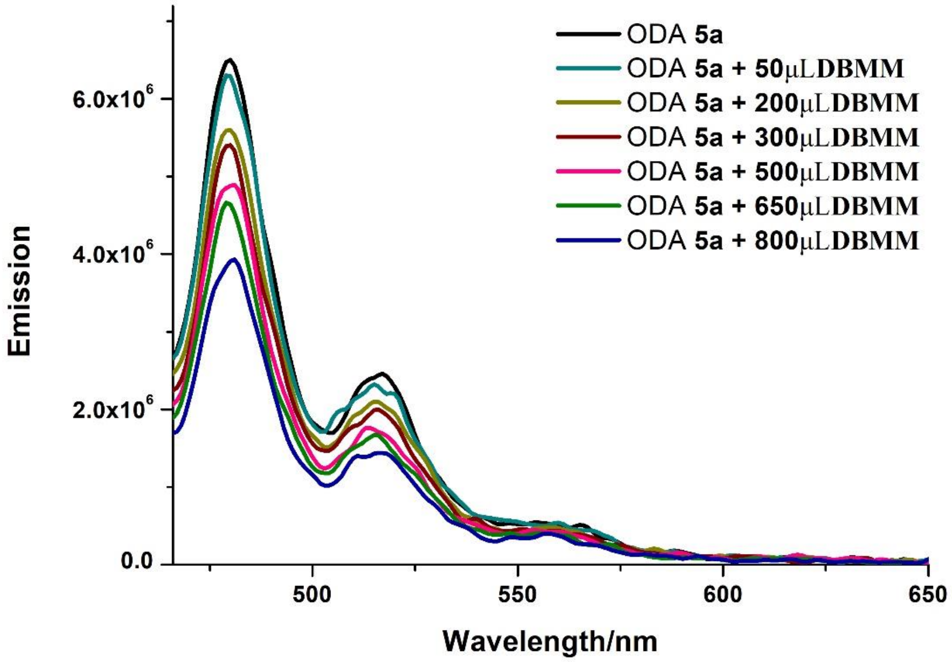

Supplementary Figure 15 | Fluorescence quenching of ODA 5a by DBMM.

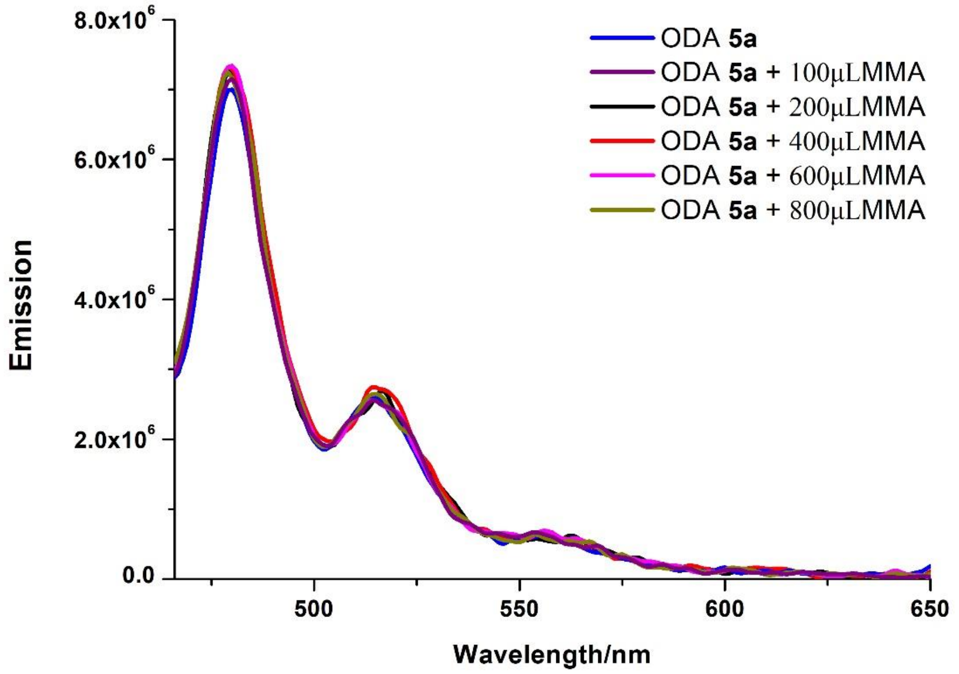

Supplementary Figure 16 | Fluorescence quenching of ODA 5a by MMA.

A

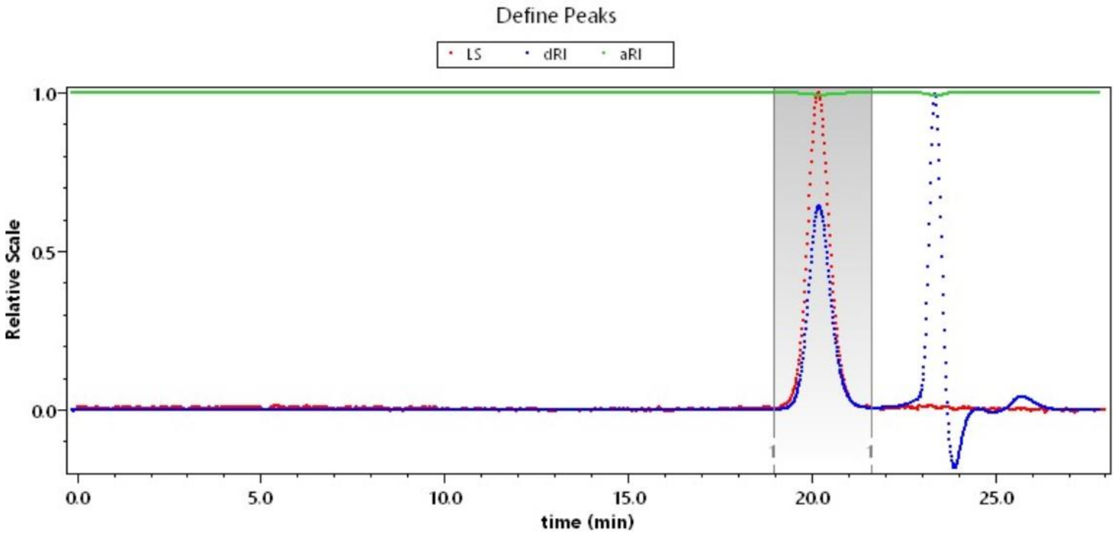

B

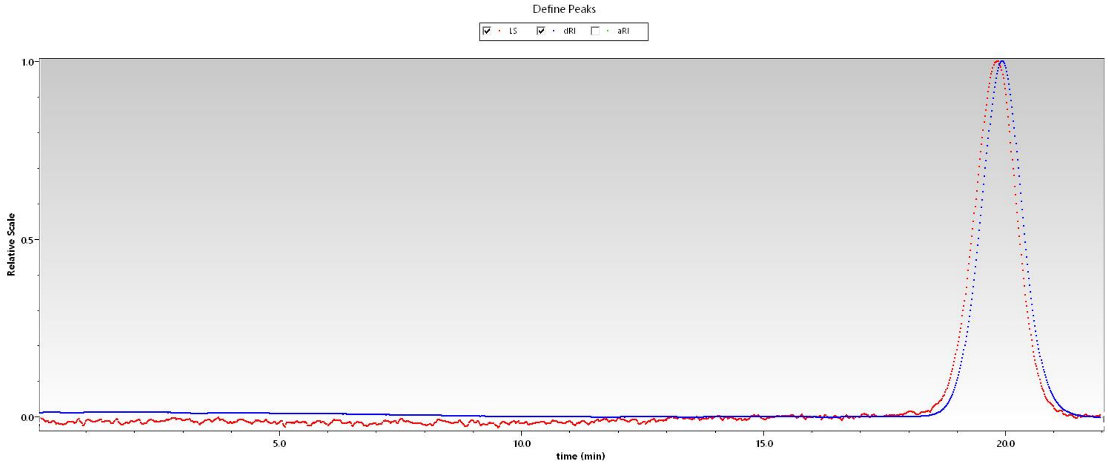

Supplementary Figure 17 | A, Example of how peaks were picked for molecular weight characterization in ASTRA 6. B, This chromatogram is of PMMA polymerized by **ODA 5d** at 10 ppm at the12-hour time point.(Table 2, Entry 5)

The color of PMMA products

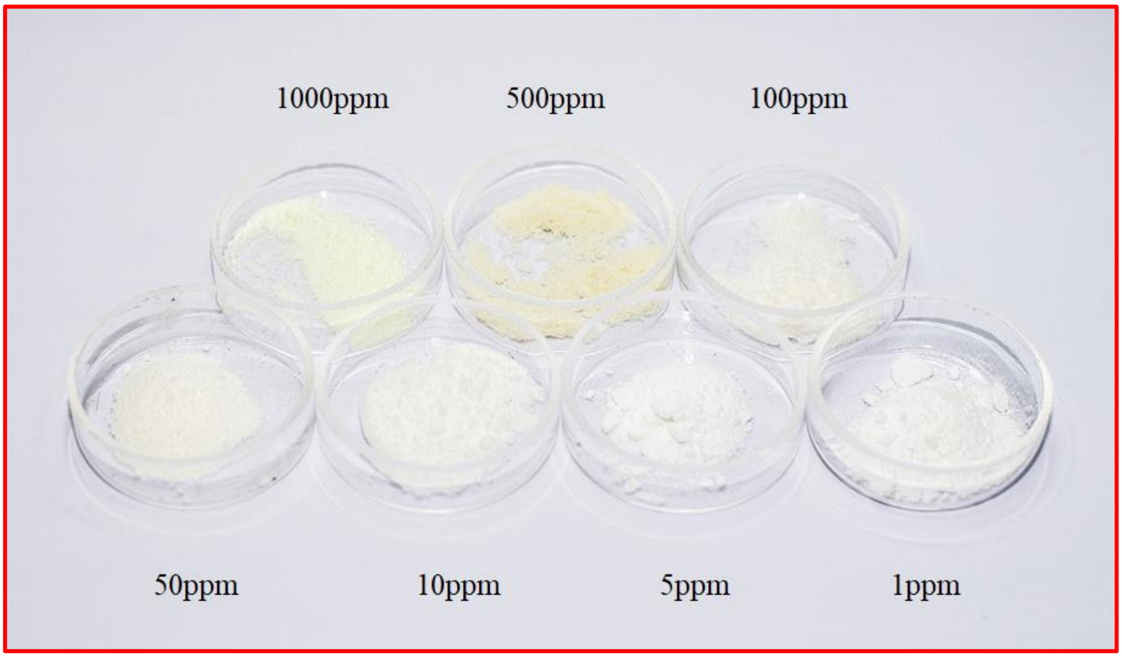

Supplementary Figure 18 | The color of PMMA products obtained with different levels of photocatalyst.

Computational detail

All of the theoretical calculations were performed in Gaussian09 package. Geometries optimization calculations were carried out by a meta-GGA hybrid functional PBE0 with 6-31G* basis set for all atoms. Vibrational frequencies were calculated analytically at the same level to obtain the thermodynamic corrections. No imaginary frequency was obtained at optimized geometries for all species. The CPCM solvation model using the self-consistent reaction field (SCRF) method with the solvents of acetonitrile was employed to account the solvent effect. The changes in Gibbs free energy are reported in the content. The redox potentials of triplet state were calculated by the energy differences of triplet states and cation radical,

$$\Delta G_{red} (PC^{+}/{}^{3}PC^{*}) = G({}^{3}PC^{*}) - G(PC^{\bullet+}),$$

with the corrections to SHE (–4.48V) and to SCE (–0.244V) in acetonitrile,[4]

$$E^{0} (PC^{+}/{}^{3}PC^{*}) = \Delta G_{red} (PC^{+}/{}^{3}PC^{*})/ 23.06 - 4.48 - 0.244, \text{ in V.}$$

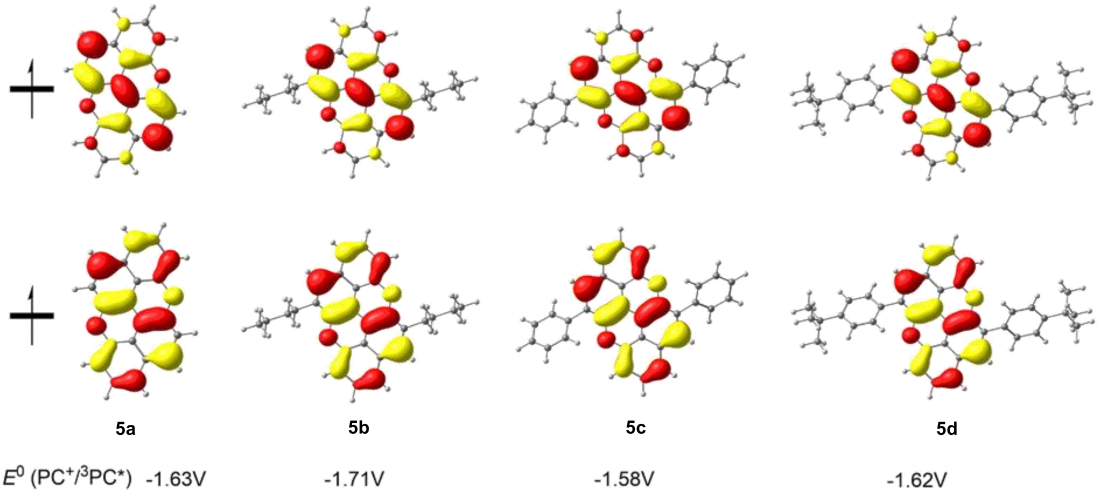

Supplementary Figure 19 | The SOMOs and redox potentials of triplet states for 5a-5d.

Computational geometries and energies

5a, R=H

```
Ground state
C    4.476932   0.833126   0.000072
C    3.424481   1.776056   0.000072
C    2.123351   1.329281   0.000039
C    1.851718  -0.061276   0.000008
C    2.908173  -1.007974   0.000010
C    4.240474  -0.523602   0.000042
C    0.513032  -0.497476  -0.000023
C    0.197993  -1.835708  -0.000048
C    1.228814  -2.795014  -0.000047
C    2.545802  -2.384587  -0.000019
C   -0.513032   0.497476  -0.000011
C   -0.197993   1.835708   0.000023
C   -1.228814   2.795014   0.000046
C   -2.545802   2.384587   0.000035
C   -2.908173   1.007974   0.000003
C   -1.851718   0.061276  -0.000020
C   -4.240474   0.523602  -0.000008
C   -4.476932  -0.833126  -0.000037
C   -3.424481  -1.776056  -0.000058
C   -2.123351  -1.329281  -0.000050
O   -1.107937  -2.259388  -0.000076
O    1.107937   2.259388   0.000034
H    5.499135   1.200697   0.000098
H    3.629380   2.841772   0.000096
H    5.066337  -1.229361   0.000044
H    3.335423  -3.130967  -0.000019
H   -3.335423   3.130967   0.000053
H   -5.066337   1.229361   0.000008
H   -5.499135  -1.200697  -0.000044
H   -3.629380  -2.841772  -0.000081
H   -0.972377   3.849928   0.000072
H    0.972377  -3.849928  -0.000068
Energies (0K) = -917.604574681
Energies (0K) + ZPE = -917.361437
Enthalpies (298K) = -917.346988
Free Energies (298K) = -917.400918

Triplet state
C    4.483797   0.819009   0.000070
C    3.455153   1.763519   0.000067
C    2.139009   1.307462   0.000036
C    1.860155  -0.060942   0.000008
C    2.898960  -1.025245   0.000011
C    4.224193  -0.549531   0.000042
C    0.491951  -0.474209  -0.000020
```

C	0.174951	-1.869239	-0.000046
C	1.181256	-2.812707	-0.000043
C	2.530149	-2.407227	-0.000015
C	-0.491951	0.474209	-0.000009
C	-0.174951	1.869239	0.000026
C	-1.181256	2.812707	0.000050
C	-2.530149	2.407227	0.000039
C	-2.898960	1.025245	0.000004
C	-1.860155	0.060942	-0.000021
C	-4.224193	0.549531	-0.000007
C	-4.483797	-0.819009	-0.000040
C	-3.455153	-1.763519	-0.000063
C	-2.139009	-1.307462	-0.000052
O	-1.140376	-2.249391	-0.000079
O	1.140376	2.249391	0.000031
H	5.513127	1.165651	0.000094
H	3.659424	2.828974	0.000089
H	5.048071	-1.257892	0.000046
H	3.312626	-3.159299	-0.000012
H	-3.312626	3.159299	0.000059
H	-5.048071	1.257892	0.000010
H	-5.513127	-1.165651	-0.000048
H	-3.659424	-2.828974	-0.000089
H	-0.924528	3.867538	0.000078
H	0.924528	-3.867538	-0.000062

Energies (0K) = -917.536315066
Energies (0K) + ZPE = -917.296855
Enthalpies (298K) = -917.282005
Free Energies (298K) = -917.337599

Cation radical

C	4.463950	0.831372	0.000057
C	3.428068	1.773552	0.000065
C	2.119218	1.320037	0.000043
C	1.845551	-0.061125	0.000012
C	2.892884	-1.012296	0.000004
C	4.216736	-0.536493	0.000026
C	0.506808	-0.486142	-0.000008
C	0.182833	-1.846278	-0.000037
C	1.207502	-2.808473	-0.000047
C	2.522259	-2.392449	-0.000027
C	-0.506808	0.486142	0.000004
C	-0.182833	1.846278	0.000035
C	-1.207502	2.808473	0.000047
C	-2.522259	2.392449	0.000029
C	-2.892884	1.012296	-0.000002
C	-1.845551	0.061125	-0.000014
C	-4.216736	0.536493	-0.000023
C	-4.463950	-0.831372	-0.000053

```
C   -3.428068  -1.773552  -0.000064
C   -2.119218  -1.320037  -0.000045
O   -1.107481  -2.239405  -0.000056
O    1.107481   2.239405   0.000052
H    5.489171   1.187012   0.000074
H    3.632702   2.838583   0.000089
H    5.042832  -1.240560   0.000021
H    3.310725  -3.139168  -0.000034
H   -3.310725   3.139168   0.000038
H   -5.042832   1.240560  -0.000015
H   -5.489171  -1.187012  -0.000068
H   -3.632702  -2.838583  -0.000088
H   -0.950796   3.861995   0.000071
H    0.950796  -3.861995  -0.000069
Energies (0K) = -917.428082074
Energies (0K) + ZPE = -917.183970
Enthalpies (298K) = -917.169601
Free Energies (298K) = -917.223952
```

5b, R=^{n}Bu

```
Ground state
C   -0.625764  -4.508834  -0.474126
C   -1.617944  -3.502706  -0.468026
C   -1.231254  -2.182011  -0.462388
C    0.143504  -1.846521  -0.461621
C    1.139397  -2.854534  -0.468288
C    0.718584  -4.208391  -0.474999
C    0.521208  -0.490490  -0.457477
C    1.844132  -0.117778  -0.456589
C    2.871215  -1.094863  -0.463049
C    2.495386  -2.426846  -0.470036
C   -0.521195   0.490514  -0.457468
C   -1.844119   0.117802  -0.456626
C   -2.871202   1.094887  -0.463070
C   -2.495373   2.426870  -0.469991
C   -1.139384   2.854558  -0.468193
C   -0.143491   1.846545  -0.461545
C   -0.718570   4.208415  -0.474836
C    0.625777   4.508859  -0.473922
C    1.617957   3.502730  -0.467843
C    1.231267   2.182035  -0.462268
O    2.204601   1.209456  -0.456565
O   -2.204588  -1.209431  -0.456667
H   -0.944016  -5.547636  -0.478607
H   -2.672857  -3.757751  -0.468210
H    1.462303  -5.000490  -0.480194
H    3.275092  -3.185485  -0.479884
```

```
H   -3.275079   3.185510  -0.479827
H   -1.462290   5.000515  -0.480015
H    0.944030   5.547660  -0.478352
H    2.672870   3.757775  -0.467993
C    4.316616  -0.680715  -0.432042
H    4.933633  -1.515824  -0.784463
C   -4.316604   0.680737  -0.432122
H   -4.933611   1.515863  -0.784520
H   -4.475777  -0.151452  -1.129567
H    4.475810   0.151508  -1.129442
C    4.788418  -0.256617   0.964033
H    4.161626   0.570202   1.323430
H    4.635879  -1.090878   1.662652
C    6.253242   0.167749   0.984843
H    6.398373   0.998417   0.280160
H    6.875207  -0.659232   0.614782
C    6.727594   0.584579   2.371695
H    6.623621  -0.238722   3.088541
H    7.780591   0.886683   2.360960
H    6.141283   1.429967   2.751249
C   -4.788446   0.256570   0.963918
H   -4.161661  -0.570262   1.323295
H   -4.635932   1.090799   1.662581
C   -6.253268  -0.167804   0.984663
H   -6.875226   0.659192   0.614623
H   -6.398373  -0.998438   0.279935
C   -6.727661  -0.584704   2.371480
H   -7.780656  -0.886815   2.360696
H   -6.141357  -1.430106   2.751012
H   -6.623718   0.238563   3.088370
Energies (0K) = -1231.73578137
Energies (0K) + ZPE = -1231.264061
Enthalpies (298K) = -1231.237951
Free Energies (298K) = -1231.319803

Triplet state
C   -0.547838  -4.523226  -0.469459
C   -1.554090  -3.554647  -0.467832
C   -1.179255  -2.212887  -0.468349
C    0.168804  -1.852058  -0.469674
C    1.195034  -2.827881  -0.470631
C    0.802314  -4.180240  -0.471112
C    0.503144  -0.463206  -0.470157
C    1.878163  -0.067193  -0.469238
C    2.899510  -1.004448  -0.473485
C    2.549858  -2.373394  -0.473601
C   -0.503134   0.463239  -0.470151
C   -1.878153   0.067226  -0.469278
C   -2.899500   1.004482  -0.473515
```

C	-2.549848	2.373427	-0.473570
C	-1.195024	2.827914	-0.470551
C	-0.168794	1.852091	-0.469607
C	-0.802304	4.180273	-0.470973
C	0.547848	4.523259	-0.469277
C	1.554100	3.554680	-0.467663
C	1.179266	2.212920	-0.468238
O	2.177578	1.272437	-0.465429
O	-2.177568	-1.272404	-0.465529
H	-0.830396	-5.572114	-0.469411
H	-2.604978	-3.824393	-0.466659
H	1.559807	-4.959433	-0.472407
H	3.346301	-3.112719	-0.480933
H	-3.346290	3.112752	-0.480893
H	-1.559798	4.959465	-0.472256
H	0.830405	5.572147	-0.469184
H	2.604987	3.824426	-0.466456
C	4.342312	-0.576802	-0.439171
H	4.962785	-1.384757	-0.846284
C	-4.342302	0.576832	-0.439257
H	-4.962767	1.384811	-0.846333
H	-4.487126	-0.292982	-1.092520
H	4.487152	0.293053	-1.092375
C	4.832436	-0.225495	0.970447
H	4.210253	0.583050	1.377548
H	4.685486	-1.092509	1.629451
C	6.297948	0.196241	0.998200
H	6.438701	1.056229	0.328492
H	6.915720	-0.614616	0.587432
C	6.784915	0.552698	2.397477
H	6.683884	-0.299858	3.079773
H	7.838733	0.852246	2.391489
H	6.204158	1.382792	2.817330
C	-4.832458	0.225434	0.970327
H	-4.210278	-0.583130	1.377394
H	-4.685533	1.092410	1.629387
C	-6.297967	-0.196317	0.998017
H	-6.915736	0.614558	0.587282
H	-6.438693	-1.056266	0.328252
C	-6.784967	-0.552867	2.397259
H	-7.838780	-0.852429	2.391223
H	-6.204209	-1.382978	2.817077
H	-6.683967	0.299649	3.079610

Energies (0K) = -1231.66569528
Energies (0K) + ZPE = -1231.197946
Enthalpies (298K) = -1231.171331
Free Energies (298K) = -1231.255630

Cation radical

C	-0.629411	-4.494701	-0.474406
C	-1.619306	-3.504215	-0.469829
C	-1.225331	-2.175851	-0.465814
C	0.140489	-1.840357	-0.465020
C	1.139664	-2.839677	-0.470054
C	0.725777	-4.184412	-0.475346
C	0.508855	-0.485337	-0.462395
C	1.853631	-0.104582	-0.460905
C	2.883574	-1.074939	-0.465438
C	2.499309	-2.404962	-0.471386
C	-0.508840	0.485360	-0.462384
C	-1.853617	0.104604	-0.460941
C	-2.883560	1.074961	-0.465458
C	-2.499294	2.404985	-0.471338
C	-1.139649	2.839699	-0.469955
C	-0.140474	1.840379	-0.464942
C	-0.725763	4.184435	-0.475179
C	0.629425	4.494724	-0.474198
C	1.619320	3.504237	-0.469643
C	1.225345	2.175874	-0.465692
O	2.186384	1.205275	-0.461957
O	-2.186370	-1.205253	-0.462058
H	-0.936514	-5.535675	-0.477825
H	-2.673724	-3.758073	-0.470061
H	1.466950	-4.977675	-0.479649
H	3.276860	-3.164760	-0.479958
H	-3.276846	3.164783	-0.479897
H	-1.466936	4.977698	-0.479464
H	0.936528	5.535698	-0.477566
H	2.673738	3.758096	-0.469841
C	4.326809	-0.658869	-0.432280
H	4.940799	-1.489056	-0.798551
C	-4.326796	0.658890	-0.432361
H	-4.940775	1.489093	-0.798613
H	-4.481117	-0.181277	-1.120457
H	4.481151	0.181328	-1.120333
C	4.798293	-0.255687	0.971084
H	4.175085	0.566960	1.346118
H	4.646989	-1.100139	1.656897
C	6.263805	0.166384	0.990521
H	6.406985	1.006039	0.296534
H	6.882479	-0.656990	0.607904
C	6.741683	0.563832	2.381678
H	6.638338	-0.268679	3.087702
H	7.795149	0.863608	2.370770
H	6.158570	1.405644	2.773593
C	-4.798323	0.255644	0.970970
H	-4.175124	-0.567017	1.345988
H	-4.647044	1.100066	1.656825

C	-6.263834	-0.166435	0.990340
H	-6.882499	0.656953	0.607739
H	-6.406986	-1.006061	0.296312
C	-6.741757	-0.563945	2.381465
H	-7.795220	-0.863728	2.370507
H	-6.158651	-1.405769	2.773364
H	-6.638443	0.268536	3.087527

Energies (0K) = -1231.56124762
Energies (0K) + ZPE = -1231.088598
Enthalpies (298K) = -1231.062531
Free Energies (298K) = -1231.145023

5c, R=Ph

Ground state

C	-0.165642	-4.550174	-0.034578
C	-1.252917	-3.648259	-0.018468
C	-1.000393	-2.295773	0.001115
C	0.331744	-1.822033	0.005436
C	1.422047	-2.726230	-0.016250
C	1.141162	-4.116028	-0.033473
C	0.570316	-0.434703	0.024953
C	1.848915	0.073663	0.026339
C	2.969796	-0.801040	0.010275
C	2.726098	-2.166914	-0.012826
C	-0.570318	0.434704	0.024940
C	-1.848917	-0.073663	0.026259
C	-2.969797	0.801041	0.010170
C	-2.726098	2.166916	-0.012888
C	-1.422046	2.726231	-0.016243
C	-0.331744	1.822034	0.005466
C	-1.141161	4.116031	-0.033421
C	0.165643	4.550176	-0.034461
C	1.252918	3.648261	-0.018327
C	1.000393	2.295774	0.001214
O	2.066813	1.428626	0.015261
O	-2.066814	-1.428625	0.015139
C	4.362788	-0.295231	0.003480
C	-4.362788	0.295231	0.003307
C	5.305415	-0.863793	-0.863715
C	6.628651	-0.432760	-0.860157
C	7.033369	0.575656	0.011446
C	6.104593	1.149217	0.878049
C	4.780887	0.721089	0.873770
C	-5.305381	0.863809	-0.863916
C	-6.628616	0.432774	-0.860421
C	-7.033368	-0.575661	0.011145
C	-6.104627	-1.149238	0.877775

C	-4.780921	-0.721107	0.873558
H	-0.378124	-5.615419	-0.048932
H	-2.277239	-4.006577	-0.021649
H	1.961596	-4.828024	-0.047257
H	3.576480	-2.843761	-0.006921
H	-3.576480	2.843763	-0.007003
H	-1.961594	4.828026	-0.047222
H	0.378126	5.615422	-0.048780
H	2.277240	4.006579	-0.021457
H	4.991862	-1.638441	-1.558711
H	7.342503	-0.882302	-1.545090
H	6.412375	1.932650	1.565391
H	4.068264	1.168909	1.558982
H	-4.991801	1.638472	-1.558882
H	-7.342441	0.882328	-1.545374
H	-6.412436	-1.932685	1.565089
H	-4.068325	-1.168940	1.558792
H	8.065741	0.914448	0.014429
H	-8.065740	-0.914455	0.014079

Energies (0K) = -1379.17533430
Energies (0K) + ZPE = -1378.769235
Enthalpies (298K) = -1378.745064
Free Energies (298K) = -1378.821956

Triplet state

C	-0.201636	4.552508	0.021065
C	-1.277195	3.662226	0.011678
C	-1.001950	2.296508	-0.007640
C	0.312683	1.833463	-0.018679
C	1.407710	2.730122	-0.002595
C	1.118509	4.108774	0.014071
C	0.539912	0.423749	-0.036653
C	1.878787	-0.080526	-0.036913
C	2.971683	0.781626	-0.024109
C	2.721013	2.177762	-0.007218
C	-0.539914	-0.423748	-0.036640
C	-1.878789	0.080526	-0.036833
C	-2.971685	-0.781625	-0.024003
C	-2.721013	-2.177762	-0.007156
C	-1.407710	-2.730122	-0.002599
C	-0.312684	-1.833463	-0.018708
C	-1.118508	-4.108775	0.014024
C	0.201637	-4.552509	0.020953
C	1.277196	-3.662227	0.011542
C	1.001950	-2.296508	-0.007734
O	2.066621	-1.434811	-0.014961
O	-2.066621	1.434812	-0.014842
C	4.369710	0.294049	-0.002732

C	-4.369711	-0.294049	-0.002558
C	5.312662	0.919260	0.826375
C	6.639556	0.500767	0.844360
C	7.053376	-0.552048	0.031131
C	6.127435	-1.180428	-0.799696
C	4.800235	-0.763537	-0.817568
C	-5.312630	-0.919283	0.826568
C	-6.639523	-0.500790	0.844617
C	-7.053375	0.552047	0.031434
C	-6.127467	1.180450	-0.799412
C	-4.800267	0.763560	-0.817348
H	-0.404366	5.619455	0.034987
H	-2.305321	4.008194	0.019404
H	1.933077	4.827771	0.024200
H	3.568534	2.855648	-0.029899
H	-3.568535	-2.855648	-0.029818
H	-1.933076	-4.827772	0.024170
H	0.404368	-5.619456	0.034844
H	2.305322	-4.008195	0.019218
H	4.995684	1.730696	1.476437
H	7.350292	0.996100	1.500592
H	6.440751	-1.997422	-1.444392
H	4.093617	-1.253313	-1.479836
H	-4.995626	-1.730737	1.476595
H	-7.350234	-0.996142	1.500864
H	-6.440808	1.997463	-1.444073
H	-4.093675	1.253353	-1.479630
H	8.089098	-0.880310	0.044132
H	-8.089096	0.880309	0.044484

Energies (0K) = -1379.10778535
Energies (0K) + ZPE = -1378.705536
Enthalpies (298K) = -1378.680931
Free Energies (298K) = -1378.759719

Cation radical

C	-0.212102	-4.534912	-0.026516
C	-1.287534	-3.638646	-0.013471
C	-1.015862	-2.279940	0.004130
C	0.311891	-1.819200	0.009586
C	1.396826	-2.724147	-0.008170
C	1.108603	-4.101763	-0.024246
C	0.553424	-0.435925	0.025779
C	1.857488	0.070641	0.026707
C	2.972800	-0.806871	0.016965
C	2.708727	-2.169360	-0.002184
C	-0.553426	0.435926	0.025765
C	-1.857489	-0.070641	0.026624
C	-2.972801	0.806872	0.016855
C	-2.708727	2.169362	-0.002250

```
C   -1.396826   2.724149  -0.008165
C   -0.311892   1.819201   0.009616
C   -1.108603   4.101765  -0.024194
C    0.212103   4.534914  -0.026395
C    1.287534   3.638648  -0.013325
C    1.015861   2.279941   0.004231
O    2.061874   1.404081   0.013582
O   -2.061876  -1.404079   0.013457
C    4.366419  -0.307382   0.004304
C   -4.366419   0.307381   0.004127
C    5.295486  -0.875485  -0.876745
C    6.618713  -0.446111  -0.882160
C    7.032091   0.555361  -0.006631
C    6.114674   1.125557   0.873883
C    4.790047   0.701503   0.879539
C   -5.295452   0.875499  -0.876948
C   -6.618678   0.446122  -0.882423
C   -7.032089  -0.555367  -0.006929
C   -6.114706  -1.125576   0.873611
C   -4.790080  -0.701520   0.879327
H   -0.422940  -5.599370  -0.039970
H   -2.314779  -3.986230  -0.017795
H    1.920065  -4.822811  -0.036553
H    3.551625  -2.854682   0.004634
H   -3.551626   2.854683   0.004548
H   -1.920064   4.822813  -0.036518
H    0.422941   5.599373  -0.039813
H    2.314779   3.986232  -0.017596
H    4.974019  -1.643675  -1.575158
H    7.325435  -0.891590  -1.576511
H    6.431557   1.902206   1.564182
H    4.087794   1.143196   1.579588
H   -4.973958   1.643702  -1.575334
H   -7.325374   0.891612  -1.576794
H   -6.431615  -1.902238   1.563884
H   -4.087853  -1.143224   1.579397
H    8.065084   0.891598  -0.010973
H   -8.065081  -0.891605  -0.011319
Energies (0K) = -1378.99798232
Energies (0K) + ZPE = -1378.590885
Enthalpies (298K) = -1378.566815
Free Energies (298K) = -1378.644014
```

5d, R=4-^{t}BuC$_6$H$_4$

```
Ground state
C   -0.612322  -4.511811  -0.040923
C   -1.605839  -3.507318  -0.027278
```

C	-1.221651	-2.186040	-0.009706
C	0.150686	-1.845760	-0.005012
C	1.146877	-2.852671	-0.024237
C	0.730858	-4.208034	-0.039375
C	0.524715	-0.488730	0.013239
C	1.847325	-0.109301	0.014394
C	2.877612	-1.089743	0.000369
C	2.499620	-2.425078	-0.019682
C	-0.524718	0.488742	0.013229
C	-1.847329	0.109313	0.014330
C	-2.877615	1.089756	0.000283
C	-2.499622	2.425090	-0.019730
C	-1.146878	2.852684	-0.024228
C	-0.150688	1.845773	-0.004986
C	-0.730859	4.208047	-0.039330
C	0.612321	4.511824	-0.040828
C	1.605837	3.507331	-0.027164
C	1.221649	2.186053	-0.009626
O	2.197467	1.217998	0.001804
O	-2.197469	-1.217986	0.001704
C	4.312278	-0.723463	-0.008517
C	-4.312280	0.723474	-0.008670
C	5.204194	-1.392406	-0.851848
C	6.563841	-1.090614	-0.849530
C	7.092230	-0.108990	-0.006356
C	6.190878	0.557030	0.837448
C	4.834291	0.263944	0.839170
C	-5.204161	1.392436	-0.852024
C	-6.563806	1.090636	-0.849778
C	-7.092228	0.108985	-0.006657
C	-6.190912	-0.557052	0.837173
C	-4.834328	-0.263957	0.838967
C	-8.578013	-0.253244	0.020189
C	-9.396617	0.580915	-0.968568
C	-8.746898	-1.737451	-0.344782
C	-9.136629	-0.011706	1.432345
C	8.578018	0.253220	0.020578
C	9.396661	-0.580913	-0.968170
C	8.746942	1.737438	-0.344326
C	9.136557	0.011619	1.432753
H	-0.928491	-5.551062	-0.053644
H	-2.660389	-3.763487	-0.030860
H	1.477400	-4.997194	-0.051212
H	3.278950	-3.182567	-0.010383
H	-3.278952	3.182580	-0.010447
H	-1.477401	4.997207	-0.051180
H	0.928490	5.551075	-0.053521
H	2.660388	3.763499	-0.030705
H	4.828475	-2.149354	-1.535810

H	7.211847	-1.634314	-1.528998
H	6.553100	1.324443	1.516773
H	4.174361	0.798601	1.514959
H	-4.828414	2.149404	-1.535949
H	-7.211784	1.634350	-1.529262
H	-6.553163	-1.324486	1.516459
H	-4.174427	-0.798628	1.514773
H	-10.449635	0.283647	-0.912665
H	-9.341943	1.651825	-0.741609
H	-9.064237	0.430667	-2.002082
H	-9.808306	-2.012338	-0.324056
H	-8.218783	-2.391917	0.356816
H	-8.362362	-1.939484	-1.351016
H	-9.037828	1.041839	1.717718
H	-10.200441	-0.275833	1.466400
H	-8.618617	-0.616011	2.184623
H	10.449680	-0.283663	-0.912199
H	9.341959	-1.651831	-0.741255
H	9.064337	-0.430620	-2.001694
H	9.808353	2.012309	-0.323534
H	8.218800	2.391884	0.357270
H	8.362461	1.939515	-1.350573
H	9.037726	-1.041936	1.718081
H	10.200372	0.275728	1.466874
H	8.618515	0.615902	2.185028

Energies (0K) = -1693.30065835
Energies (0K) + ZPE = -1692.667558
Enthalpies (298K) = -1692.632292
Free Energies (298K) = -1692.733568

Triplet state

C	0.632468	4.512864	-0.002862
C	1.618914	3.524565	0.004645
C	1.215753	2.190931	0.023141
C	-0.136996	1.854663	0.035027
C	-1.142085	2.851101	0.020287
C	-0.723769	4.196093	0.004736
C	-0.497229	0.473002	0.053028
C	-1.878135	0.098886	0.051796
C	-2.884968	1.060543	0.038846
C	-2.501884	2.426504	0.024910
C	0.497231	-0.472999	0.053017
C	1.878137	-0.098883	0.051723
C	2.884969	-1.060540	0.038747
C	2.501883	-2.426502	0.024852
C	1.142085	-2.851099	0.020291
C	0.136996	-1.854661	0.035054
C	0.723768	-4.196092	0.004780
C	-0.632469	-4.512862	-0.002759

C	-1.618914	-3.524563	0.004770
C	-1.215753	-2.190928	0.023227
O	-2.193866	-1.232045	0.028642
O	2.193866	1.232047	0.028533
C	-4.321974	0.708908	0.011833
C	4.321975	-0.708908	0.011663
C	-5.207537	1.430044	-0.796634
C	-6.568690	1.136720	-0.822077
C	-7.110109	0.112281	-0.040280
C	-6.218457	-0.603377	0.772568
C	-4.860447	-0.317999	0.801788
C	5.207501	-1.430068	-0.796823
C	6.568654	-1.136746	-0.822335
C	7.110108	-0.112285	-0.040592
C	6.218493	0.603397	0.772275
C	4.860484	0.318021	0.801565
C	8.597733	0.243349	-0.045467
C	9.403415	-0.643903	-0.997881
C	8.770205	1.705934	-0.488359
C	9.169041	0.071988	1.371706
C	-8.597734	-0.243353	-0.045078
C	-9.403456	0.643863	-0.997493
C	-8.770226	-1.705955	-0.487905
C	-9.168980	-0.071935	1.372112
H	0.935448	5.555830	-0.015946
H	2.675164	3.771772	-0.003930
H	-1.466630	4.988948	-0.004140
H	-3.280935	3.181980	0.049062
H	3.280935	-3.181978	0.048987
H	1.466629	-4.988946	-0.004112
H	-0.935450	-5.555828	-0.015814
H	-2.675165	-3.771770	-0.003760
H	-4.824575	2.223638	-1.433466
H	-7.208106	1.722229	-1.474757
H	-6.590458	-1.403763	1.407455
H	-4.211677	-0.891169	1.456412
H	4.824511	-2.223679	-1.433616
H	7.208040	-1.722275	-1.475026
H	6.590523	1.403802	1.407122
H	4.211745	0.891211	1.456202
H	10.457936	-0.347889	-0.968504
H	9.347806	-1.701526	-0.715591
H	9.060248	-0.545801	-2.034136
H	9.832841	1.976985	-0.490445
H	8.250128	2.398303	0.182133
H	8.378536	1.857464	-1.500760
H	9.070156	-0.965672	1.710414
H	10.233815	0.334235	1.383197
H	8.658966	0.714511	2.097223

```
H  -10.457976   0.347849  -0.968062
H   -9.347837   1.701496  -0.715244
H   -9.060331   0.545723  -2.033758
H   -9.832864  -1.977003  -0.489937
H   -8.250125  -2.398298   0.182595
H   -8.378598  -1.857526  -1.500315
H   -9.070083   0.965739   1.710774
H  -10.233754  -0.334183   1.383661
H   -8.658872  -0.714428   2.097633
 Energies (0K) = -1693.23292330
Energies (0K) + ZPE = -1692.603500
Enthalpies (298K) = -1692.567872
Free Energies (298K) = -1692.670598

Cation radical
C    0.643708   4.493926  -0.027201
C    1.629006   3.499237  -0.016117
C    1.229171   2.172462   0.000978
C   -0.136534   1.840509   0.007487
C   -1.130352   2.844631  -0.008546
C   -0.712266   4.188385  -0.023688
C   -0.509299   0.486738   0.023482
C   -1.855943   0.107631   0.023907
C   -2.883520   1.087178   0.015445
C   -2.489163   2.418318  -0.000889
C    0.509302  -0.486746   0.023470
C    1.855946  -0.107639   0.023834
C    2.883523  -1.087186   0.015348
C    2.489165  -2.418326  -0.000947
C    1.130354  -2.844640  -0.008542
C    0.136536  -1.840517   0.007512
C    0.712267  -4.188394  -0.023645
C   -0.643708  -4.493934  -0.027099
C   -1.629004  -3.499246  -0.015994
C   -1.229170  -2.172470   0.001063
O   -2.186668  -1.200614   0.008803
O    2.186669   1.200606   0.008696
C   -4.316968   0.724284   0.000293
C    4.316970  -0.724292   0.000129
C   -5.197819   1.395855  -0.851938
C   -6.556629   1.093888  -0.859366
C   -7.091178   0.113625  -0.018099
C   -6.198285  -0.552439   0.834688
C   -4.841789  -0.261782   0.846982
C    5.197786  -1.395885  -0.852121
C    6.556594  -1.093913  -0.859618
C    7.091177  -0.113623  -0.018404
C    6.198319   0.552462   0.834404
C    4.841825   0.261799   0.846768
```

```
C    8.577087   0.247085  -0.002752
C    9.387038  -0.586279  -0.999179
C    8.742522   1.732001  -0.366989
C    9.145442   0.002843   1.405197
C   -8.577091  -0.247068  -0.002365
C   -9.387081   0.586266  -0.998785
C   -8.742559  -1.731997  -0.366535
C   -9.145376  -0.002765   1.405602
H    0.954848   5.533570  -0.040061
H    2.684675   3.747648  -0.021523
H   -1.451251   4.983515  -0.034512
H   -3.262484   3.181223   0.009669
H    3.262486  -3.181231   0.009594
H    1.451252  -4.983524  -0.034483
H   -0.954848  -5.533579  -0.039929
H   -2.684674  -3.747656  -0.021354
H   -4.816787   2.150419  -1.535529
H   -7.199112   1.636402  -1.544502
H   -6.566766  -1.317426   1.512758
H   -4.190525  -0.793046   1.534084
H    4.816727  -2.150470  -1.535674
H    7.199050  -1.636444  -1.544767
H    6.566828   1.317470   1.512435
H    4.190589   0.793080   1.533884
H   10.440404  -0.289462  -0.950451
H    9.333853  -1.657476  -0.773320
H    9.047435  -0.433704  -2.029972
H    9.804279   2.005387  -0.354885
H    8.221413   2.385889   0.340327
H    8.349716   1.935735  -1.369620
H    9.047104  -1.050738   1.690341
H   10.209849   0.264896   1.429989
H    8.635436   0.607831   2.162329
H  -10.440447   0.289460  -0.949997
H   -9.333875   1.657470  -0.772968
H   -9.047527   0.433650  -2.029589
H   -9.804319  -2.005371  -0.354370
H   -8.221425  -2.385863   0.340782
H   -8.349803  -1.935774  -1.369177
H   -9.047013   1.050826   1.690700
H  -10.209784  -0.264807   1.430456
H   -8.635339  -0.607730   2.162733
Energies (0K) = -1693.12382254
Energies (0K) + ZPE = -1692.489881
Enthalpies (298K) = -1692.454676
Free Energies (298K) = -1692.556460
```

Comparison Tables

Supplementary Table 9. Previous reports on catalyst loading (or catalyst residue) in polymers prepared by ATRP.[a]

Entry	Monomer	Catalyst/Initiator System	Catalyst Residue	Reaction Temp (°C)	Reference
1	MMA	ODA **5d**/DBMM	0.05ppm-10ppm	25, Light	This work
2	MMA	core-substituted diaryl dihydrophenazine/DBMM	5-100ppm	25, Light	*Macromolecules* ***2019****, 52, 747-754*
3	MMA	4DP-IPN/DBMM	0.5 ppm-100ppm	25, Light	*Nat. Catal.* **2018**, 1, 794-804
4	MMA	*fac*-Ir(ppy)$_3$, EBPA, PEGMA	<16 ppm	25, Light	*Polym. Chem.* **2018**, 9, 584
5	MMA	EBP/Ph-benzoPTZ	500-1000ppm	25, Light	*Chem. Eur. J.* **2017**, 23, 5972-5977
6	MMA	4CZ-IPN/EBP	5-40ppm	25, Light	*Macromol. Rapid Commun.* **2017**, 38, 1600461
7	MMA	pyrene or anthracene/EBP	100-30000ppm	25, Light	*Macromolecules* ***2016****, 49, 7785-7792*
8	MMA	EBP/PMDETA/Eosin Y or Erythrosin B	500ppm	25, Light	*Polym. Chem.* **2016**, 7, 6094–6098
9	MMA	diphenyl dihydrophenazine/EBP	1000ppm	25, Light	*Science* **2016,** 352, 1082-1086
10	MMA	Diffusion-regulated phase transfer catalyst(DRPTC) in an aqueous-organic biphasic system $CuBr_2$/EBrPA/tris(2-pyridylmethyl)amine	6-26 ppm	75	*Macromol. Rap. Comm.* **2015**, 36, 538-546
11	MMA	PTH/EBP	1000ppm	25, Light	*J. Am. Chem. Soc.* ***2014****, 136, 16096-16101*
12	MMA	*fac*-Ir(ppy)$_3$/EBP	50ppm	25, Light	*Angew. Chem. Int. Ed.* **2012**, 51, 8850-8853
13	MMA	CuBr/4,4'-bis(RfCH2OCH2)-2,2'-bpy complexes	19.3 ppm	90	*J. Appl. Polym. sci.* **2008**, 110, 2531
14	MMA	Ionic liquid catalyst TEDETA anchored on an imidazolium-based ionic liquid	50-100 ppm	60	*Macromolecules* **2005**, 38, 5921
15	MMA	Silica supported catalyst,Cabosil-CuBr/dMBpy	37 ppm	90	*Journal of catalysis*, 232, **2005**, 276-294
16	MMA	Thymine anchored on silica gel support and 2,6-diaminopyridine functionalized with a catalyst, CuBr(TEDETA) complex	39 ppm	90	*J. Polymer Science Part A.* **2004**, 42, 22-30

17	MMA	Crosslinked diphosphinopolystyrene supported NiBr2(TPP)2/EBriB, TPP ligand	34 ppm	90	*Chem. Commun.* **2004**, 640-641
18	MMA	Tris(2(dioctadecylamino)ethyl)amine CuBr/EBriB	200 ppm	70	*J. Am. Chem. Soc.* **2004**, 126, 7764-7765
19	MMA, St DMAEMA	JandJael ligend, CuBr/EBriB	331-463 ppm	60-100	*Macromolecules* **2003**, 36, 3111-3114
20	MMA	CuBr/Preciption ligands/EBriB	<70 ppm	90	*Macromolecules* **2002**, 35, 4849-4851
21	MMA	CuBr/PE25-PEG4-TEDETA/MBPA	8-35 ppm	80	*Macromolecules* **2001**, 34, 8603-8609
22	MMA, BA	Dual immobilized/soluble hybrid catalyst system, CuBr/dNbpy, CuBr/PMDETA, and CuBr/Me6TREN	15 ppm	90	*Macromolecules* **2001**, 34, 5099-5102
23	MMA and MA	The new two component catalyst system CuBr/PS-bpy and a soluble catalyst, Cubr$_2$/Me$_6$TREN	20 ppm	80	*Macromolecules* **2001**, 34, 5099
24	MMA	Pentakis-N-(4,4,5,5,6,6,7,7,8,8,9,9,10,10,11,11,11-heptadecafluoroundecyl)-1,4,7-triazaheptane, CuBr, EBriB	880 ppm	90	*J. Am. Chem. Soc.* **2000**, 122, 1542-1543
25	MMA	Amphiphilic block copolymer with bipyridine ligands ML1/CuBr/EBriB	100 ppm	60	
26	MMA	3-Aminopropyl-functionalized silica complexed with RuCl$_2$(PPh$_3$)$_3$/EBriB	1000 ppm	90	*Macromolecules* **1999**, 32, 4769-4775
27	MMA	Crosslinked polystyrene Merrifield resin supported catalysts, CuBr-s-TREN/EBPA	120 ppm	90	*Macromolecules* **1999**, 32, 2941-2947

[a] The values with metal catalysts before 2017 were summarized according to the Supplementary Table 7 in *Nat. Catal.* **1**, 794-804 (2018).[5]

Supplementary Table 10. Comparison with other OPCs in O-ATRP

	This work	Science 352, 1082 (2016)	Macro. Rapid Commun. 38, 1600461 (2017)	Nat. Catal. 1, 794 (2018)	Macromolecules 52, 747 (2019)	Angew. Chem. Int. Ed. 59, 3209 (2020)
**The best catalyst (framework)**	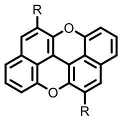	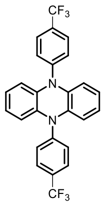	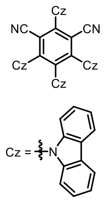	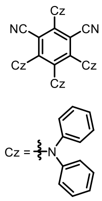	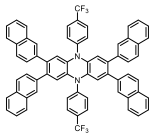	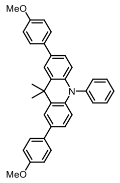
**Structural character**	*O-*	*N-*	*N-*	*N-*	*N-*	*N-*
**Absorption maximum λ_{max}**	~450 nm visible light	< 400 nm in ultraviolet	< 400 nm in ultraviolet	< 400 nm in ultraviolet	< 400 nm in ultraviolet	< 400 nm in ultraviolet
**Design**	New principle Non-CT	Charge Transfer (CT)	Charge Transfer (CT)	Charge Transfer (CT)	Charge Transfer (CT)	Charge Transfer (CT)
**E^0 ($PC^{\bullet+}/{}^1PC^*$) E^0 ($PC^{\bullet+}/PC$)**	-1.84 V +0.80 V	-1.80 V +0.29 V	/ /	-1.41 V +1.01 V	-1.84 V +0.38 V	-1.73 V +0.71 V
**Cat. loading for Đ <1.20**	10 ppm	1000 ppm 33.8% conv.	Not reached Đ >1.50	Not reached Đ >1.37	100 ppm*	/
**Lowest cat. loading**	0.05 ppm	200 ppm	15 ppm	0.5 ppm	5 ppm	/
**Cat. loading (sunlight)**	10 ppm 51% conv/7h	1000 ppm 34% conv/7h	/	/	/	/
**Control exp. w/o initiator**	<5% conv. with 500 ppm cat	/	41.6% conv with 500 ppm cat.	/	/	/
**Results for the polymerization of challenging monomers (with the best catalyst shown above)**						
**Styrene**	Đ 2.13/10 ppm Đ 1.99/50 ppm	0%conv/1000ppm	/	/	0% conv/50 ppm **Đ 2.41**/500 ppm†	/
***n*-Butyl acrylate**	**Đ 1.37**/10 ppm **Đ 1.11**/1000 pmm	**Đ 1.62**/1000 ppm	/	/	**Đ 2.34**/500 ppm	**Đ 2.13**/100 pmm **Đ 1.53**/1000 pmm

-The results summarized here were from polymerizations typically carried out in batch at a $[MMA]_0/[Initiator]_0$ ratio of 100:1 except that in *Nat. Catal.* (200:1).[5] Dispersity were determined by GPC with PMMA standards unless otherwise specified. "/" means not reported or not applicable. cat. = catalyst. exp. = experiment. λ_{max}: only considering the absorption profile above 310 nm. Đ values in purple were determined by GPC coupled with MALS. *Đ 1.09 by GPC(PMMA) equal to Đ ~1.20 by GPC(MALS) is employed. † With catalyst **3a** rather than the best catalyst **3c** in ATRP of MMA.

Supplementary References

1. Allen, R. D., Long, T. E. & McGrath, J. E. Preparation of high purity, anionic polymerization grade alkyl methacrylate monomers. *Polym. Bull.*, **15**, 127–134 (1986).

2. Kamei, T., Uryu, M. & Shimada, T. Cu-catalyzed aerobic oxidative C–H/C–O cyclization of 2,2'-binaphthols: practical synthesis of PXX derivatives. *Org. Lett.* **19**, 2714–2717 (2017).

3. Treat, N. J., Sprafke, H., Kramer, J. W., Clark, P. G., Read de Alaniz, J., Fors, B. P. & Hawker, C. J. Metal-free atom transfer radical polymerization. *J. Am. Chem. Soc.* **136**, 16096–16101 (2014).

4. Kelly, C. P., Cramer, C. J. & Truhlar, D. G. Single-ion solvation free energies and the normal hydrogen electrode potential in methanol, acetonitrile, and dimethyl sulfoxide. *J. Phys. Chem. B* **111**, 408−422 (2007).

5. Singh, V. K., Yu, C., Badgujar, S., Kim, Y., Kwon, Y., Kim, D., Lee, J., Akhter, T., Thangavel, G., Park, L. S., Lee, J., Nandajan, P. C., Wannemacher, R., Milián-Medina, B., Lüer, L., Kim, K. S., Gierschner, J. & Kwon, M. S. Highly efficient organic photocatalysts discovered via a computer-aided-design strategy for visible-light-driven atom transfer radical polymerization. *Nat. Catal.* **1**, 794−804 (2018).

6. Liu, D., Dai, L., Lin, X., Chen, J.-F., Zhang, J., Feng, X., Müllen, K., Zhu, X. & Dai, S. Chemical Approaches to Carbon-Based Metal-Free Catalysts, *Adv. Mater.* **31**, 1804863 (2019).
